# Mutualism supports biodiversity when the direct competition is weak

**DOI:** 10.1038/ncomms14326

**Published:** 2017-02-24

**Authors:** Alberto Pascual-García, Ugo Bastolla

**Affiliations:** 1 Centro de Biologia Molecular ‘Severo Ochoa’ CSIC-UAM Cantoblanco, 28049 Madrid, Spain

## Abstract

A key question of theoretical ecology is which properties of ecosystems favour their stability and help maintaining biodiversity. This question recently reconsidered mutualistic systems, generating intense controversy about the role of mutualistic interactions and their network architecture. Here we show analytically and verify with simulations that reducing the effective interspecific competition and the propagation of perturbations positively influences structural stability against environmental perturbations, enhancing persistence. Noteworthy, mutualism reduces the effective interspecific competition only when the direct interspecific competition is weaker than a critical value. This critical competition is in almost all cases larger in pollinator networks than in random networks with the same connectance. Highly connected mutualistic networks reduce the propagation of environmental perturbations, a mechanism reminiscent of MacArthur’s proposal that ecosystem complexity enhances stability. Our analytic framework rationalizes previous contradictory results, and it gives valuable insight on the complex relationship between mutualism and biodiversity.

## Abstract (web summary)

Theoretical ecologists have made disparate predictions regarding the relationship between mutualism and stability. Here, Pascual-García and Bastolla develop a theoretical framework showing that model mutualistic interactions promote structural stability when competition is weaker than a threshold depending on network architecture.

Which properties of ecosystems enhance their stability against environmental perturbation, favouring the maintenance of biodiversity, is a key question of theoretical ecology. In this context, the concept of complexity had a preeminent role^1^, starting from the inspiring proposal by MacArthur that the complexity of ecosystems favours their stability^2^, disputed by the model of May^3^. Currently, ecologists prefer to focus on ecosystem architecture rather than complexity^4^,^5^, but the essence of the question remains the same.

MacArthur argued that a perturbation would be more easily damped, thus diminishing its negative effect on the rest of the system, when the number of interactions is large because there is a large number of possible paths for the flux of nutrients^2^. Nevertheless, his argument was not based on any explicit dynamical model. On the other hand, May’s classical analysis of the complexity-stability relationship adopted population dynamics equations close to a feasible equilibrium point (that is, all abundances are positive), finding that the probability that the system is dynamically stable against perturbations of the species abundances rapidly vanishes as the number of species and interactions increases.

May’s approach has been extremely influential, but it has limitations^6^. First, as recognized by May himself, environmental perturbations can modify all parameters of the population dynamics and not just species abundances, thus the dynamical stability analysis, which assumes that parameters are fixed, addresses a limited spectrum of perturbations. Second, the constraints imposed by feasibility may even guarantee dynamical stability, such as in the case of diagonally stable interaction matrices.

A complementary approach is based on the analysis of structural stability, which quantifies how the system responds to changes of the parameters, such as growth rates or interaction strengths. Although the structural stability of ecosystems against environmental perturbations is arguably a main determinant of the maintenance of biodiversity, its study is less common in theoretical ecology than in other fields of computational biology^7^, with few exceptions^8^,^9^,^10^,^11^,^12^.

In recent years, both because of new empirical data and new theoretical approaches, much of the theoretical work on ecosystem stability addressed mutualistic networks of plants and pollinators and plants and seed dispersers^4^,^9^,^10^,^12^,^13^,^14^,^15^,^16^,^17^,^18^,^19^. Despite intense work, these studies disagree on the effect of mutualism on persistence. Whereas some indicated that, in some conditions, mutualism increases the persistence of model ecosystems^9^,^10^,^12^,^14^, others reached the opposite conclusion^15^, and different studies highlighted either nestedness^9^,^12^ or connectance^14^,^15^ as the network property that most influences persistence. In particular, we and coworkers showed analytically that fully connected mutualistic networks favour structural stability by reducing the effective competition between species in the same group (plants or pollinators), and that the more nested a sparse mutualistic network is, the weaker its effective competition^9^. On the basis of these results, we argued in^9^ that nested mutualistic networks increase structural stability. However, this conclusion is not valid in general, as we show in the present paper. Subsequently, the idea that nested mutualistic interactions tend to favour stability seemed to be confirmed by the simulations by Thebault and Fontaine^14^, but it was later criticized by James *et al*.^15^, whose simulations showed that mutualistic interactions tend to be deleterious for ecosystem persistence, and that the network property that better correlates with persistence is connectance and not nestedness. Our work started as a technical response to this paper^10^, but it later became more complex. Meanwhile, Rohr *et al*. published a paper based on similar ideas that seemed to show that nested mutualistic interactions are always good for the persistence of ecosystems^12^, something that is contrasted by the results that we present here.

Here we propose a novel framework that allows analytically predicting and numerically testing the structural stability of model ecosystems. Our analysis starts observing that, if the interaction matrix has a mathematical property called diagonal stability, every feasible equilibrium is globally dynamically stable^20^, so that structural stability can be measured as the maximum perturbation of parameters that maintains feasibility. It is interesting to note that the effective competition is strongly related with global stability, since diagonal stability of the effective competition matrix is a necessary and almost sufficient condition for global stability of the full interaction matrix^21^. We found that structural stability defined in this way can be predicted based on three quantities: (1) the effective interspecific competition^8^, which expresses the competition between animals or between plants that results both from their direct interaction and from their interaction with species in the other group, (2) the propagation of perturbations, which describes how the perturbations of the intrinsic growth rates affect the productivity of each species and (3) the vulnerability of the unperturbed system, which measures the deficit in productivity of the species that can be most affected by perturbations. These quantities are differently influenced by the connectance and the nestedness of mutualistic networks, providing analytical understanding of which topological properties are more relevant in different regimes of parameters. Whereas the first quantity is directly related with the dynamical stability, the second quantity is strongly related with the ideas put forward by MacArthur^2^. In this way, the analysis of structural stability encompasses the arguments proposed by both MacArthur and May, reconciling their perspectives.

## Results

### Numerical assessment of structural stability

Following previous work^9^, we model two groups of *S*^(P)^ plants and *S*^(A)^ animal species. We only report the equations for plants, since those for animals can be obtained interchanging the superscripts P and A. Species interact through within-group fully connected competition matrices 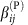 of Lotka–Volterra (LV) type and between-group mutualistic interactions 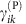 that are non-zero only if there is a link between *i* and *k* in the mutualistic network and that saturate for large abundance^13^,^22^,^23^


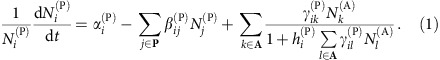


where 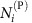 denotes the abundance of species *i*, 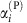 is its intrinsic growth rate, and 1/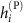 is the maximum mutualistic growth rate. In this work, abundances are measured in units such that the intraspecific competition is 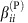
=1 for all species. The ratio between interspecific and intraspecific competition is *ρ*<1.

We set , where denotes the scale of mutualistic interactions. We distinguish between strong mutualism, when at the equilibrium point the mutualistic interactions are close to saturation (large ), and weak mutualism otherwise. Since the saturation term is proportional to the mutualistic degree, species with large degree may be in the strong-mutualistic regime while species with small degree are in the weak regime. The signs of the intrinsic growth rates distinguish between facultative mutualism if the α_i_ are positive and obligatory mutualism if they are negative.

**Figure 1:**
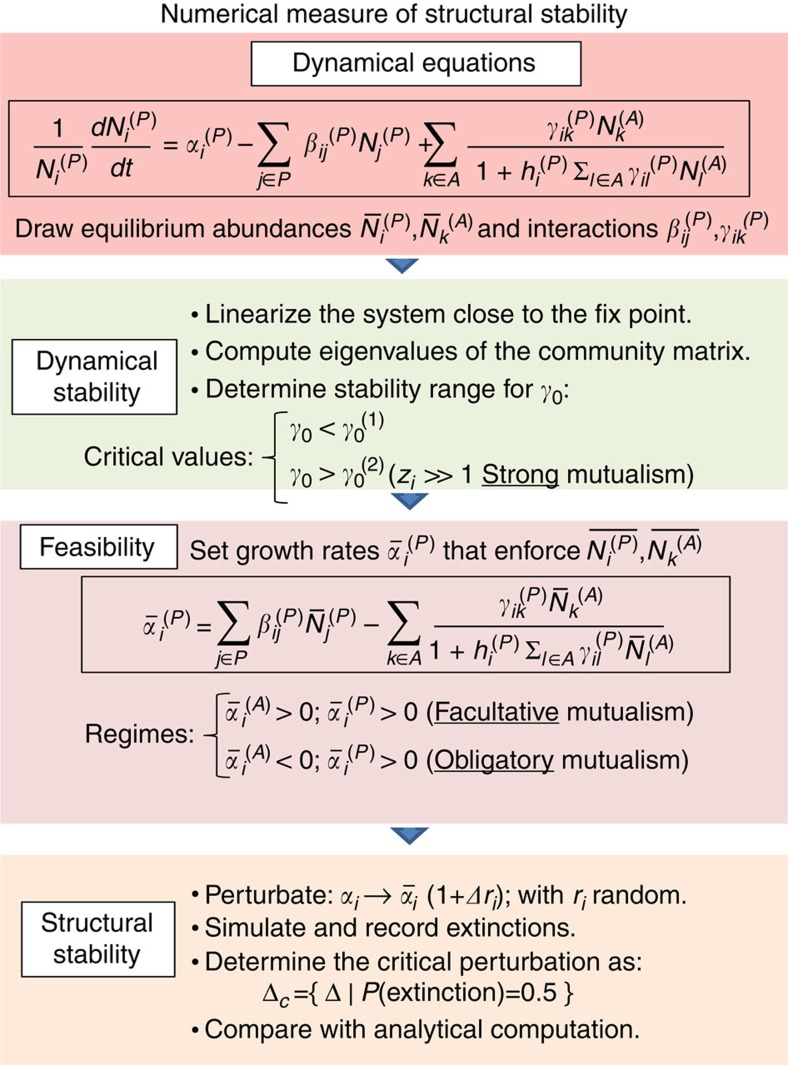
Scheme of the numerical measurement of structural stability. Each box describes a step of the simulation.

Given a realization of the ecological interactions *β*_*ij*_ and *γ*_*ik*_, if we randomly draw the intrinsic growth rates *α*_*i*_ we obtain with high probability unfeasible equilibria (that is, some abundance is not positive), in particular if the number of links per species (degree) of the mutualistic network is broadly distributed. Therefore, we determined the *α*_*i*_ by imposing that the equilibrium is feasible and dynamically stable and studied its structural stability against perturbations of the growth rates through the step-wise procedure described below. For details, see Methods and Fig. 1.

1. Abundances and interactions: We draw positive unperturbed equilibrium abundances 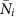 >0 and interactions *β*_*ij*_ and *g*_*ik*_ such that *γ*_*ik*_=*γ*_0_ *g*_*ik*_.
2. Dynamical Stability: We choose the scale *γ*_0_ of mutualistic interactions such that the equilibrium is dynamically stable for all networks. For facultative mutualism, we find two critical values 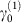 < 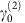 (Supplementary Fig. 1) such that the equilibrium is stable both for *γ*_0_< 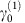 and for *γ*_0_> 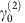 . The dependence of 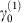 , 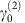 on network architecture is reported in Supplementary Fig. 3. For obligatory mutualism strong for animals and weak for plants, the equilibrium is stable for all values of *γ*_0_, as predicted below.
3. Feasibility: We set the unperturbed growth rates *α*_*i*_ in such a way that the vector 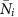 is a fixed point of equation (1). Notably, if the variance of the abundances is small the *α*_*i*_ that we obtain are negatively correlated with the mutualistic degree, which can be interpreted as a trade-off between the number of interactions and their metabolic cost. On the other hand, the unperturbed growth rates *α*_*i*_ are identically distributed if the mutualistic interactions are absent and the competition network is fully connected, as assumed here.
4. Structural Stability: We perform 100 independent simulations in which we randomly perturbate all growth rates by a relative amount Δ. The percentage *e* of simulations where at least one species becomes extinct versus Δ follows a sigmoidal curve with vertical tangent close to the point Δ_c_ at which *e*=0.5, providing a natural measure of structural stability.

Structural stability is often defined as the volume of the space of growth-rate parameters compatible with the coexistence of all species. However, a direct measure of this volume for a large number of species requires exponentially long computations. Our proposed measure is much simpler and it is ecologically relevant, since it simulates the effect of environmental perturbations, so that, the larger Δ_c_, the more likely is that the system maintains coexistence against perturbations.

### Simulated regimes and numerical results

We studied either facultative mutualism, or mutualism that is facultative for plants and obligatory for animals. A natural way to achieve this regime is to choose larger equilibrium abundances for plants than for animals, and large saturated mutualistic growth rates 1/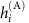 for animals (see Methods). For simplicity, we did not consider obligatory mutualism for plants, or mixed situations in which mutualism is obligatory for some animals and facultative for others. We studied meta-parameters that correspond to several combinations of regimes (facultative or obligatory, weak or strong mutualism, weak or strong competition), described in Table 1.

For all parameters sets, we simulated a representative set of 125 mutualistic networks characterized through two properties (Supplementary Fig. 5): (1) The connectance, *κ*=<*d*^(P)^/*S*^(A)^>=<*d*^(A)^/*S*^(P)^>, where 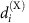 represents the degree of species *i* in group X and (2) The ratio between the second and the first moment of the degree distribution, 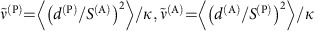. The quantities 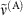 and 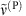 are related to the fraction of shared links, and they are well approximated by the nestedness defined in (ref. 9) (see Methods), so we can transfer predictions from one quantity to the other.

**Figure 2:**
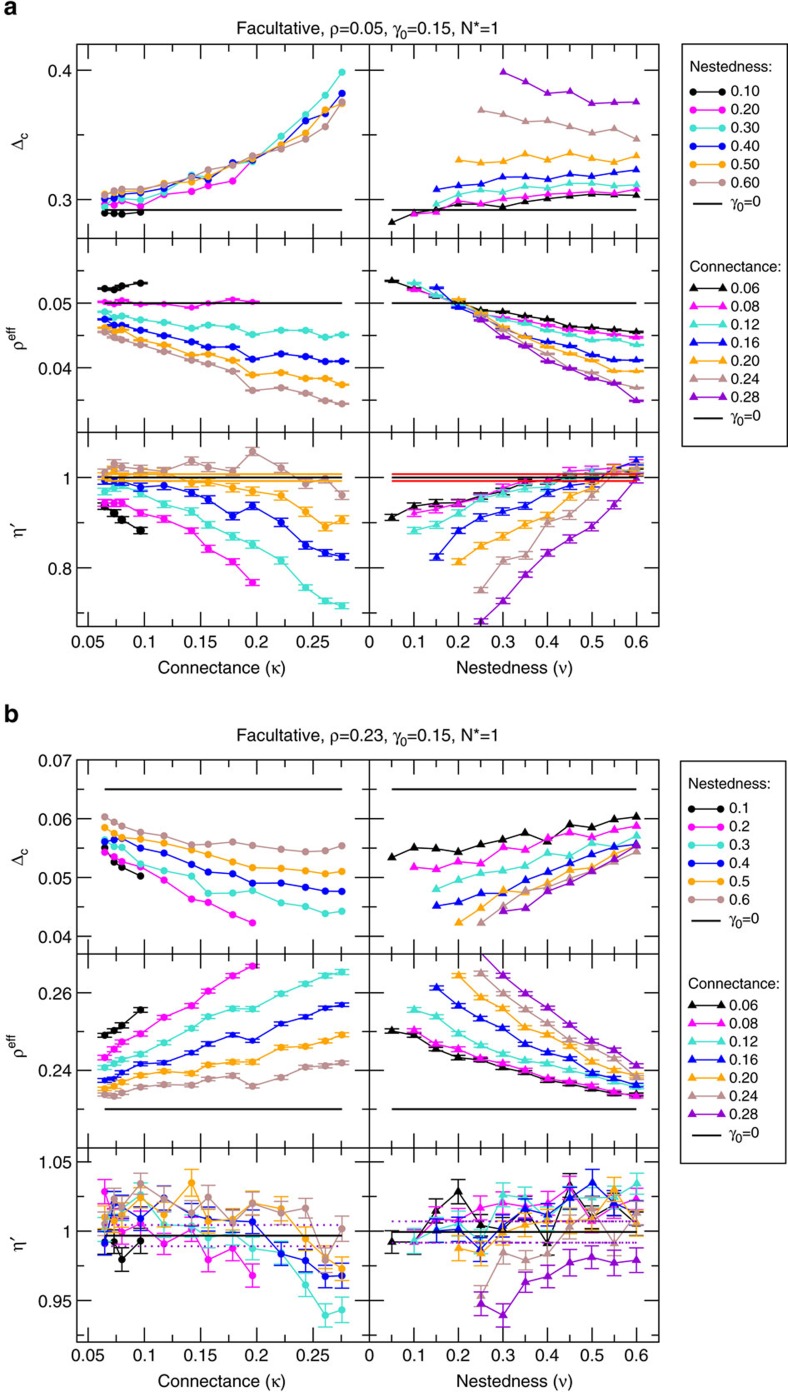
Structural stability and related quantities versus network properties. We represent here two regimes of facultative weak mutualism. Each point represents one of the 125 artificial networks. Error bars quantify the s.d. of the mean over 50 realizations of the parameters and the ecological dynamics. The horizontal lines refers to the absence of inter-species interactions, that is, pure direct competition. Note that the effective competition is smaller than the direct competition for small *ρ*=0.05 (**a**) while the opposite occurs for large *ρ*=0.23 (**b**).

Figure 2 (top panels) shows that mutualistic networks affect structural stability in a complex way in different regimes of parameters. The figure shows two sets of networks in the facultative weak-mutualistic regime characterized by small (*ρ*=0.05) and large (*ρ*=0.23) interspecific competition (regimes A and C). Additional regimes are presented in the Supplementary Note 2. For reference, we plot as an horizontal line the structural stability of a competitive system without mutualism. Mutualism tends to increase the structural stability for *ρ*=0.05, but it tends to decrease it for *ρ*=0.23. Moreover, for *ρ*=0.05 structural stability is positively correlated with the connectance and its correlation with nestedness depends on whether the connectance is large or small, while for *ρ*=0.23 structural stability is negatively correlated with the connectance and positively correlated with the nestedness.

### The effective competition framework

**Figure 3:**
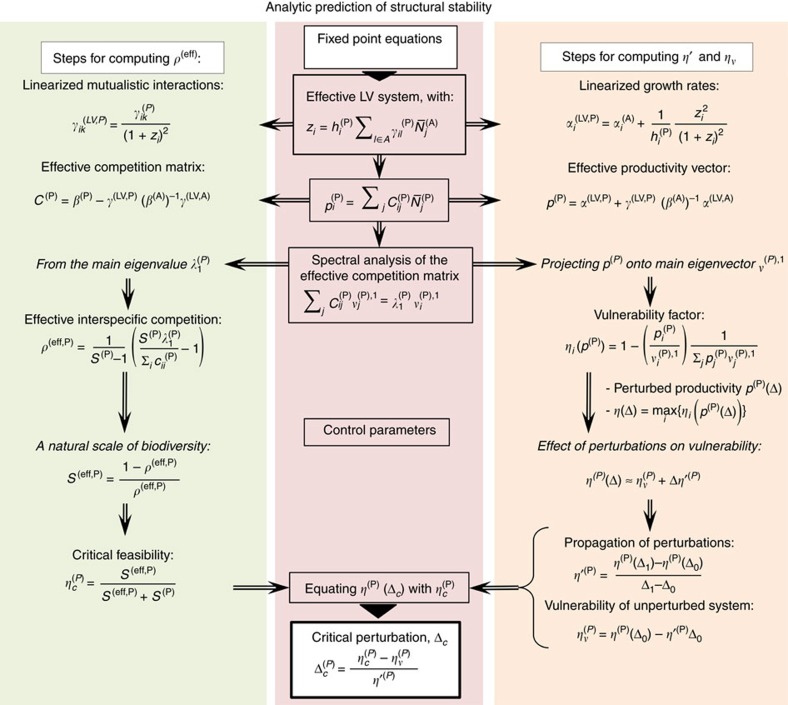
Flux of the analytical prediction of structural stability. The steps to compute the interspecific effective competition parameter *ρ*^eff^ and the propagation of perturbations *η*′ are represented in the left and right column, respectively.

To interpret the above results, we adapted the effective competition framework, originally developed in^8^ for LV equations. The scheme of the computation is summarized in Fig. 3 and described in this and the next subsections. First of all, we linearize the dynamical equations close to the equilibrium point 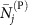, 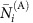, obtaining an equivalent LV system with effective growth rates 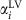 and effective interactions 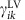 (see Methods).

From this linearized system, we separate the equilibrium equations as 
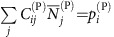
 for plants and equivalent for animals^8^ (see Methods). The matrix ***C***^(P)^ represents the effective competition between plant species, which includes their indirect interactions through animals. Similarly, ***p***^(P)^ is the effective productivity vector, accounting for intrinsic growth rates and ecological interactions. Although in this work we focus on mutualism, this formalism allows to deal with predation simply by changing the sign of the interactions between plants and animals.

The effective competition framework allows reducing the complexity of a two-guilds system containing competitive and mutualistic (and/or predatory) interactions into an equivalent competitive system for a single guild. While the complete interaction matrix has both positive and negative components, we expect that the components of ***C***^(P)^ are positive, so that the Perron–Frobenius theorem implies that its main eigenvector ***v***^(*P*),1^ has only positive components^24^. However, positivity of all components of ***C***^(P)^ is not necessary since ***v***^(*P*),1^ is also positive if the non-diagonal components are negative and small in absolute value, for instance larger than −1/(*S*^(P)^−1) in units such that *C*_*ii*_=1. The main eigenvector ***v***^(*P*),1^ yields the optimal productivity vector that guarantees positivity of the equilibrium abundances of all species^8^.

### Effective competition hinders structural stability

Here and in the following, we omit for simplicity the superscripts A and P whenever no ambiguity arises. The main eigenvalue of ***C*** allows computing the effective interspecific competition *ρ*^eff^ (see Methods) measured in units of the intraspecific competition. It was shown in^8^ that the smaller *ρ*^eff^, the larger the structural stability. Given a productivity vector *p*_*i*_, we define the vulnerability of species *i* as 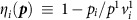, where 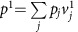 is the projection of the productivity vector onto ***v***^1^. Sufficient condition for feasibility is max_*i*_{*η*_*i*_(***p***)}≤*η*^*c*^≡*S*^eff^/(*S*^eff^+*S*), which we call feasibility condition^8^. Here *S* is the number of species and *S*^eff^=(1−*ρ*^eff^)/*ρ*^eff^ is a natural scale of biodiversity. The larger *ρ*^eff^ and *S*, the more difficult it is to fulfil feasibility, consistent with the classic result of May^3^. In particular, for 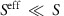 (large *S* or *ρ*^eff^≈1) the condition only holds if the productivity vector is almost parallel to ***v***^1^.

### Competition critically determines the influence of mutualism

Since nestedness tends to reduce *ρ*^eff^ (ref. 9), it was suggested that nested mutualistic networks favour structural stability. Nevertheless, we found here that the effect of mutualism on the effective competition depends on the value of the direct competition parameter *ρ*. A simple computation, reported in the Methods section, predicts that, for small *γ*_0_, the effective competition is


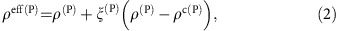


with *ξ*^(P)^>0. In particular, *ρ*^eff(P)^ is smaller than the value *ρ*^(P)^ found for pure competition only if the direct interspecific competition *ρ*^(P)^ is smaller than the critical value *ρ*^c(P)^, otherwise mutualistic interactions enhance *ρ*^eff(P)^. This point is a central result of our study that was not noted in previous works^9^,^12^. In the weak-mutualistic regime (small *γ*_0_, small 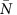 and small connectance, regimes A and C) *ρ*^c(P)^ is given by


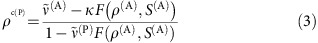


(see Methods), where 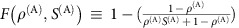. Fully connected networks (*κ*=1, 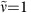) have *ρ*^c^=1, thus for these networks mutualism always reduces *ρ*^eff^ as previously shown in ref. 9.

Equation (3) shows that *ρ*^c(P)^ increases with the nestedness of the mutualistic network (which is well approximated by 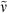) and, as a consequence of equation (2), *ρ*^eff^ decreases with nestedness, as confirmed by Fig. 2 (middle panels). Since *ξ*^(P)^ is proportional to *κS*^(A)^*γ*_0_^2^, *ρ*^eff^ decreases with the connectance and with *γ*_0_ if *ρ*<*ρ*^*c*^ but increases otherwise, consistent with Fig. 2. Finally, the networks for which *ρ*^eff^=*ρ*, hence *ρ*^c^=*ρ* (horizontal lines in Fig. 2), have an almost constant nestedness, that is, *ρ*^c^ depends weakly on connectance when *κ* is small.

### Connected networks reduce the propagation of perturbations

Next, we quantify how much the mutualistic networks amplify or damp the perturbations of the intrinsic growth rates, which in our model represent the environmental variability. We numerically compute the vulnerability *η*(Δ)=max_*i*_{*η*_*i*_(***p***(Δ))}, where *p*_*i*_(Δ) is the productivity vector obtained by perturbing the growth rates ***α*** with a random perturbation of amplitude Δ. For large Δ, *η*(Δ)≈*η*_*v*_(***p***^0^)+Δ*η*′(***p***^0^) is approximately linear. We call *η*′(***p***^0^) propagation of perturbations and *η*_*v*_(***p***^0^) unperturbed vulnerability, which depend on the unperturbed productivities ***p***^0^ (see Methods). The larger *η*′, the more destabilizing are the effects of perturbations.

It can be shown analytically that *η*′ decreases with the number of mutualistic interactions (see Methods). Thus, we expect that connected networks decrease *η*′ and enhance structural stability, consistent with our numerical results (Fig. 2 third row). From the same figure we see that *η*′ increases with nestedness, as expected, since networks with equal connectance and larger nestedness have more heterogeneous degree distribution, hence they contain more species with few links that are more vulnerable to perturbations.

### Analytical prediction of structural stability

Finally, we predict the structural stability for the effective competition system. We tested numerically that, under random perturbations, the feasibility condition becomes almost necessary, that is, it is extremely unlikely to extract feasible systems that violate it (A. Pascual-García *et al*., unpublished). Thus, we predict Δ_c_ as the maximum value of Δ such that the condition holds, which satisfies the equation *η*(Δ_c_)=*η*^c^, yielding the result


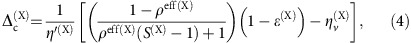


where X indicates either plants or animals, 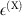
=0 for facultative mutualism and for animals, while for plants in obligatory mutualism 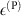 is related with the minimum plant abundance that maintains the most vulnerable animal species (see Methods). The critical perturbation Δ_c_ is the smaller between 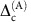 and 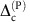.

**Figure 4:**
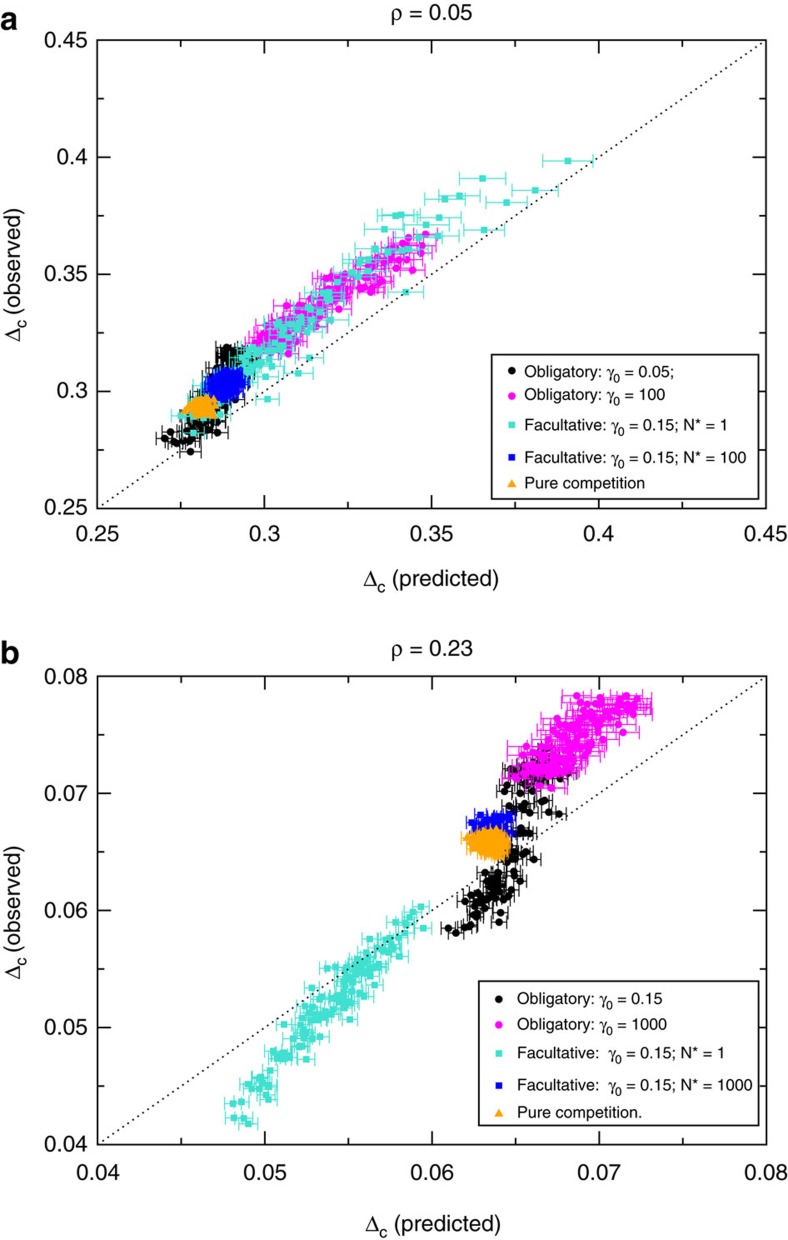
Observed versus predicted structural stability. Structural stability Δ_c_ is measured as the relative perturbation of intrinsic growth rates such that the probability that at least one species becomes extinct is 0.5. Each point represent one different mutualistic network, and each set of points with the same symbol represent different mutualistic regimes. (**a**) direct competition parameter *ρ*=0.05. (**b**) *ρ*=0.23. The equilibrium abundances are extracted from an uniform distribution in (0.85, 1.15). For each parameter regime, each point represents one of the 125 artificial networks and error bars represent the s.e. of the mean over 50 realizations of the parameters and the ecological dynamics.

Thus, according to our theory, *η*′, *ρ*^eff^ and *η*_*v*_ are sufficient to predict the structural stability Δ_c_. The good agreement between theory and simulations is shown in Fig. 4 for eight different mutualistic regimes (A to H). Additional regimes are studied in the Supplementary Note 3.

### Understanding the influence of network architecture

**Figure 5:**
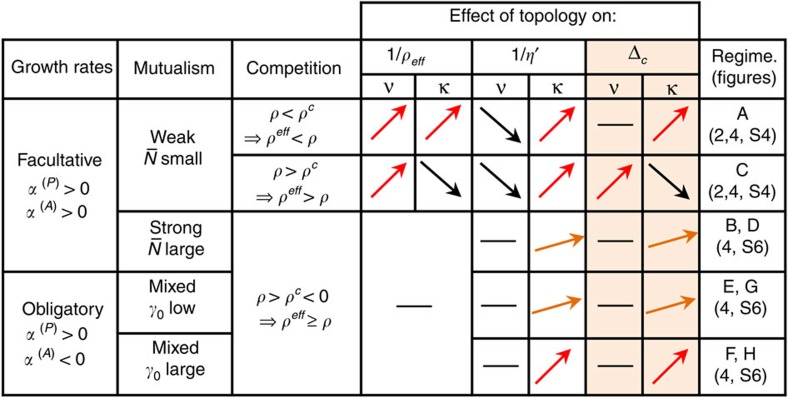
Graphical summary of the studied regimes. The first three columns describe the regime, in the next six column arrows indicate the influence of connectance *κ* and nestedness *ν* on 1/*ρ*^eff^, 1/*η*′ (the inverse variables are used because they are positively related with structural stability) and Δ_c_.

The influence of the network architecture on structural stability, summarized in Fig. 5, is mediated through the quantities *η*′, *ρ*^eff^ and *η*_*v*_. We analyse here the situation in which the unperturbed vulnerability *η*_*v*_ is small. This requires that the variance of the abundances is small in the units that we are using (see Discussion). The influence of *η*_*v*_ is studied in the Supplementary Note 3.

In the facultative strong-mutualistic regime (B and D) and in the obligatory regimes (E to H) we expect that *ρ*^c^<0 except for fully connected networks, see equation (25) in Methods, thus *ρ*>*ρ*^c^. In these regimes, *ρ*^eff^ is slightly larger than *ρ* and it is insensitive to network properties (Supplementary Fig. 6), therefore the main determinant of structural stability is the connectance through its diminishing influence on *η*′.

In contrast, in the facultative weak-mutualistic regimes (A and C) we find *ρ*^c^>0. In all cases, the nestedness decreases *ρ*^eff^ but it also increases *η*′. The connectance decreases *ρ*^eff^ for *ρ*<*ρ*^c^ but increases it for *ρ*>*ρ*^c^, as predicted by Equation (3), and decreases *η*′ in all cases (Fig. 2). These different behaviours generate a complex picture, explaining some discrepancies found in the literature. In regime A, the connectance decreases both *η*′ and *ρ*^eff^, having a strong positive influence on structural stability (Fig. 2a, *ρ*=0.05). The influence of the nestedness is modest, since it reduces *ρ*^eff^ but it increases *η*′ (Fig. 2a), with contradictory effects on structural stability. In regime C the connectance increases *ρ*^eff^, while it has only a small effect on *η*′, resulting in a negative influence on structural stability (Fig. 2b, *ρ*=0.23), while the nestedness has a weak effect on *η*′ but it has a strong diminishing effect on *ρ*^eff^, thus enhancing structural stability.

### Critical competition of mutualistic networks

Equation (3) expresses the critical *ρ*^c(P)^ for plants as a function of the properties of the mutualistic network and of the direct competition parameter *ρ*^(A)^. By iterating these equations for both plants and animals until a fixed point (*ρ*^c(P)^, *ρ*^c(A)^), we can determine the intrinsic critical competition of the mutualistic network such that whenever both *ρ*^(P)^<*ρ*^c(P)^, *ρ*^(A)^<*ρ*^c(A)^ hold, mutualistic interactions reduce the interspecific competition. We verified that the fixed point is independent of the initial values (*ρ*^(P)^, *ρ*^(A)^).

**Figure 6:**
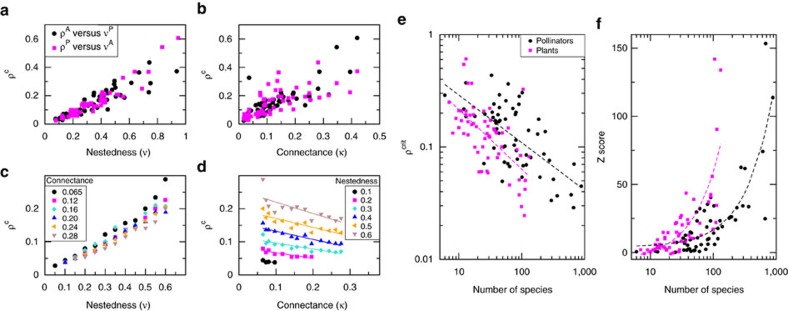
Critical direct competition *ρ*^c^ above which mutualistic interactions increase the effective competition. *ρ*^c^ was computed in the weak-mutualistic regime by iterating equation (3) for 59 real pollinator networks. *ρ*^c^ versus nestedness (**a**) and connectance (**b**) for real networks and for simulated networks (**c**,**d**) with *S*^(A)^=46, *S*^(P)^=47. Each curve of simulated networks is for fixed connectance (**c**) and nestedness (**d**). (**e**) *ρ*^c^ versus the number of species for real networks. (**f**) Z–score of *ρ*^c^ with respect to random networks with the same connectance.

We show in Fig. 6 the results of this computation for 59 pollinator networks downloaded from the Web of Life dataset^25^. The larger *ρ*^c^ is, the more mutualism favours biodiversity. As expected from Equation (3), *ρ*^c^ increases with the nestedness (Fig. 6a,c). It also increases with the connectance (Fig. 6b), but this happens because in observed networks nestedness and connectance are correlated. In contrast, when nestedness is fixed, *ρ*^c^ is negatively related with the connectance, in agreement with Equation (3) (Fig. 6d). Furthermore, *ρ*^c^ decreases with the number of species (Fig. 6e), which can be explained both by Equation (3) and by the negative relation between the nestedness and *S*.

We then computed the Z-scores of *ρ*^c^ of observed mutualistic networks with respect to random networks with the same connectance and number of species. Notably, these Z-scores are positive, which implies that *ρ*^eff^ is lower for real than for random networks, they are significant in most cases, and they increase with the number of species (Fig. 6f), that is, larger mutualistic networks have *ρ*^c^ that diverges more from that of a random network.

## Discussion

Here we developed a new framework to assess the structural stability of mutualistic model ecosystems. The good agreement between the analytical results and the numerical experiments indicates that the complexity of the full interaction matrix with (*S*^(A)^+*S*^(P)^)^2^ elements can be summarized by six quantities, namely the effective interspecific competition *ρ*^eff^, the propagation of perturbations *η*′ and the vulnerability of the unperturbed system of both plants and animals, which suffice to accurately predict structural stability. This simplification greatly facilitates the understanding of this model ecosystem by investigating how these quantities depend on the network architecture and parameters.

Our results underscore the critical role of the direct interspecific competition, showing that mutualistic interactions decrease the effective competition only when the direct competition is weak, otherwise they increase it. This analysis can be extended to predatory interactions, and we can analytically see that their dependence on competition is the opposite of the one of mutualism, since predatory interactions reduce the effective competition for strong direct competition and increase it otherwise (see Supplementary Note 4). These results may have far reaching consequences for the design and control not only of model ecological systems, but also of economical systems, if the analogy remains valid. They suggest that a cooperative association enhances stability only if the competition is weak, otherwise it has destabilizing effects.

For facultative mutualism we found that in most real pollinator networks the critical competition is larger than it would be in an equivalent random network with the same connectance, meaning that mutualism reduces the effective competition and favours structural stability even for larger direct competition. The critical competition parameter decreases with the number of species, essentially because of the decrease of the connectance, however its Z–score increases, meaning that large mutualistic networks are more different from random networks. There is currently no empirical data that can establish whether real mutualistic ecosystems act above or below the critical competition, but we can distinguish two kinds of situations. In the first situation the connectance of the competition network is constant, as assumed in the present model. If this hypothesis is correct, our results suggest that mutualism favours biodiversity in small ecosystems, which are characterized by large *ρ*^c^>*ρ*, but it hinders it in large ecosystems. Alternatively, the connectance of the competition network may decrease with the number of species, as it happens for the mutualistic network, so that *ρ* decreases with the number of species. In this situation weak facultative mutualism may be always beneficial for structural stability and biodiversity.

Furthermore, the effective competition framework reveals an inverse relation between *ρ*^eff^ and abundances. In fact, for large *S*, the feasibility condition requires that the productivity vector is almost parallel to the main eigenvector of the effective competition matrix ***v***^1^ (ref. 8). In this case, the equilibrium abundances of a feasible and diverse ecosystem are 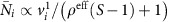. This allows integrating in the same theoretical framework the recent proposal that nested mutualistic networks may be the result of positive selection to maximize the abundance of individual species^19^. Finally, dynamical stability is also negatively correlated with *ρ*^eff^ (Supplementary Note 1; Supplementary Fig. 2), as we analytically predict in the Methods section, thus it is positively correlated with structural stability (Supplementary Fig. 4). Therefore, when *ρ*^eff^ is reduced, structural stability, dynamical stability and species abundance increase at the same time.

Besides the effective competition, the other quantity that has a strong influence on structural stability is the rate at which environmental perturbations propagate into perturbations of the effective productivities. We found that this rate decreases for increasing connectance, which in this way exherts a positive influence on structural stability. This mechanism is highly similar to the one proposed by MacArthur to argue that ecosystem complexity favours stability^2^. However, for strong direct competition the connectance increases the effective competition (which, in this regime, is more relevant than the propagation of perturbations), and it has a negative influence on structural stability—a result closer to the perspective of May^3^.

Third, structural stability is influenced by the vulnerability of the unperturbed system, *η*_*v*_(***p***^0^), which is small if the abundances of the unperturbed system are narrowly distributed, thereby enhancing structural stability. To interpret this result, note that we adopt units such that the intraspecific competition *β*_*ii*_, related with the carrying capacity, is the same for all species of animals and plants. To achieve this, we must use different abundance units for each species. From the original units 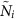, for instance number of individuals per square kilometre, we obtain a mathematically equivalent dynamical system with *β*_*ii*_≡1 through the change of units 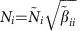, 
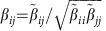
, 
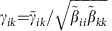
, 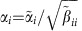. Therefore, our model ecosystem with narrowly distributed *β*_*ii*_ and *N*_*i*_ may arise from broadly distributed 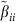 and 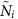 in units of number of individuals. If the unperturbed vulnerability is not small, the structural stability drops to zero when *η*_*v*_(***p***^0^)≈*η*^*c*^. This is why in the main text we focus our attention on the small *η*_*v*_ regime, as we did in our previous work^10^. The same was assumed by Rohr *et al*.^12^, who chose the *α*_*i*_ that maximize the structural stability computed as in ref. 8, which implies *η*_*v*_(***p***^0^)≈0. However, their method is only valid when the mutualistic growth rates are linear, and it cannot be reliably applied with the saturation term.

Our results help rationalizing why previous works obtained qualitatively different relationships between network architecture and stability^9^,^10^,^12^,^13^,^14^,^15^,^16^,^17^,^18^. These discrepancies have mainly two origins: The types of perturbation that challenge the model ecosystems, and the parameter regimes that are simulated.

First of all, the dynamical stability does not hold without the biologically realistic saturation term *h*_*i*_>0 in the functional response, in particular for species with large degree, for systems with *γ*_0_>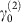, and in the obligatory weak-strong regime. Previous studies that analysed dynamical stability without taking into account saturation^26^,^27^ reached conclusions that are only valid in the special case *h*_*i*_≡0 (ref. 13). Therefore, it is crucial to adopt the saturation term.

James *et al*.^15^ extracted the growth rates from the same distribution for all mutualistic species, whereas in feasible systems the growth rates are negatively correlated with the mutualistic degree. Their method produced unfeasible equilibria with much higher probability for mutualistic systems than for competitive systems, which explains why they found that mutualism hinders biodiversity. Imposing feasibility, as we do here and in ref. 10, provides a less biased comparison of ecological interactions or network properties. Moreover, feasibility constrains ecological parameters: it predicts a trade-off between the number of mutualistic interactions and the intrinsic growth rate and, for obligatory mutualism, it requires that the ratio between plant and animal abundances must be *N*^(P)^/*N*^(A)^>2·10^5^, consistent with the empirical estimate *N*^(P)^/*N*^(A)^≈5 × 10^6^ (ref. 28) (see Methods).

Okuyama and Holland^13^ did not consider interspecific competition (*ρ*=0). In this case, the effective competition framework predicts *η*^*c*^=1, and the equilibrium is feasible if all species have positive producivity *p*_*i*_>0, which always happened since they chose positive growth rates. Consistently, their system did not feature any extinction. However, feasibility becomes a severe problem for *ρ*≠0.

Also Thébault and Fontaine^14^ considered *ρ*=0, but in their study the growth rates were negative so that feasibility was violated when *p*_*i*_<0. In this case we expect a strong correlation between survival probability and number of links, as also found by James *et al*.^15^, since more links decrease the probability that p_i_<0, Consistently, they found that connectance favours persistence and nestedness has a weak and negative effect. They also found a positive effect of nestedness and connectance on resilience, consistent with the fact that, in the studied weak competition regime *ρ*<*ρ*^*c*^, these variables tend to decrease *ρ*^eff^ with a positive effect on dynamical stability. This also rationalizes the similar result found by Okuyama and Holland^13^ and similar results in a posterior work of the same group^17^.

Bastolla *et al*.^9^ adopted the same framework discussed here, but they considered fully connected networks for which *ρ*<*ρ*^*c*^=1, which explains the conclusion that nested mutualistic interactions favour biodiversity. The work by Rohr *et al*.^12^ is most similar to the framework that we used here and in a previous work^10^. However, apparently they did not compare the structural stability of mutualistic and competitive systems, therefore they could not detect the change of regime that happens at the critical competition coefficient *ρ*^*c*^, probably also because they did not analyse small *ρ*. Their reported result that nestedness favours structural stability is consistent with our findings.

Valdovinos and coworkers showed that the stability of pollination networks can be enhanced by adaptive foraging (AF), a strategy through which pollinators devote more effort to more rewarding plants. Whereas in our model shared mutualistic interactions indirectly reduce the interspecific competition, in the regime of the model that they investigate and in the absence of AF shared resources induce an indirect competition between pollinators (for resources) and between plants (for pollinator effort). AF reduces this indirect competition^16^, thereby enhancing stability. Therefore, in the absence of AF nestedness increases competition and has a negative impact on stability that is reversed by AF (ref. 18). It would be interesting to investigate more formally the correspondence between their model and our simpler model in the framework of effective competition. We expect that in their model shared mutualistic interactions with AF reduce the effective competition similarly to how they do in our model in which AF is not represented, and that the critical competition has a role in their model as well.

Despite key questions still lack a definite answer, the theoretical framework that we presented brings analytic understanding on a dispute that has engaged several research groups. Our framework may pave the way to predict the effect on structural stability of changes in natural ecosystems, such as the introduction or removal of species and their interactions. Depending on the parameter regime, changes that modify either the connectance or the nestedness may have a more severe effect. In conclusion, computational modelling of complex ecological systems, though still in its infancy, identifies different regimes that may enhance the predictive power. These are hopefully good news for increasingly important conservation strategies, in particular for the critical role of pollination services^29^.

## Methods

### Networks descriptors

Each network is described by its adjacency matrix for plants, *a*_*ik*_, whose transpose is the adjacency matrix for animals. The degree of a species, 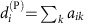 and analogous for animals, is its number of mutualistic interactions, whose average determines the connectance 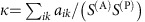. Nestedness *ν* is defined as proposed in ref. 9,


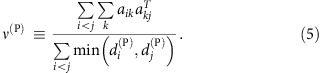


namely the average number of shared links between species of the same group normalized to one. From the above equation it appears that the nestedness of plants is correlated with the second moment of the degree of animals, in such a way that for small connectance more broadly distributed degrees imply larger nestedness: 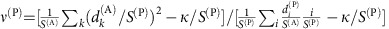, where species are ranked inversely to the number of links.

### Construction of mutualistic networks

The mutualistic networks studied in this paper have *S*^(A)^=46 animal and *S*^(P)^=47 plant species, as found in a field study at Cainama, Venezuela^30^ that was selected because (i) it has a similar number of plant and animal species and (ii) has connectance *κ*=0.07 close to the median of the entire set of mutualistic networks (*κ*=0.09) and nestedness *ν*=0.15. We used the observed degrees of plants and animals of this network, ***d***^P,obs^ and ***d***^A,obs^, as a starting point to generate 125 random networks with different combinations of connectance and nestedness as follows. First, we randomly draw links with probability 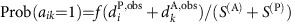. Networks drawn with *f*=1 have on the average the same connectance as the observed network and a degree distribution that interpolates between the observed network and a random network with uncorrelated links. The connectance is modified through the parameter *f*. For each value of connectance, we obtain different values of the nestedness by applying the algorithm by Medan *et al*.^31^ that swaps links maintaining the degree. Each swapping is selected with a Metropolis criterion that enforces the target nestedness. Convergence is typically achieved after 20,000 swaps.

### Parameterizing the dynamical equations

For each network, we model the multi-species population dynamics through equation (1)^9^,^10^,^15^. We generate the fully connected direct competition matrix as


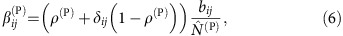


where the metaparameters *ρ*^(P)^, *ρ*^(A)^∈[0, 1] set the direct interspecific competition measured in units of intraspecific competition, 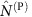 and 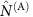 set the scale of the carrying capacity for plant and animal populations, respectively, *δ*_*ij*_ is Kronecker’s delta and *b*_*ij*_ are dimensionless random numbers uniformly distributed in [1−*δ*_*b*_, 1+*δ*_*b*_]. We parameterize mutualistic interactions as


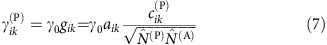


where *γ*_0_ measure the strength of mutualism with respect to competition and the dimensionless parameters *c*^(P)^ are uniformly distributed between 1−*δ*_*c*_ and 1+*δ*_*c*_ if *a*_*ik*_=1 and they are zero if *a*_*ik*_=0. The maximum mutualistic growth rates 1/*h*_*i*_ are chosen equal to 1/*H*^(P)^ for plants, and 1/*H*^(A)^ for animals. We then draw the equilibrium abundances as 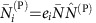. The results presented in the main text were obtained with *e*_*i*_ uniformly distributed in [1±*δ*_*N*_], with *δ*_*N*_=0.15, while in Supplementary Note 3 we present results obtained with log-normal distributions with larger variance. We can transform the dynamical equations into the units discussed in the main text, 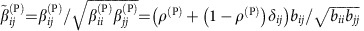 (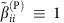), 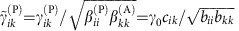 and 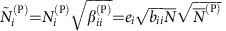. The rescaled interaction matrices only depend on the metaparameters *ρ*^(A)^, *ρ*^(P)^ and *γ*_0_. The positive metaparameters 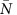, 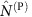 and 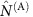 influence whether the system is in the strong mutualistic regime (mutualistic interactions are close to saturation). In most simulations we considered the dimensionless parameter 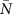
=1, but in regimes B and D we used 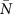
=100 to set the system in the strong-mutualistic regime while maintaining the dynamical stability.

### Obligatory and facultative mutualism

Unperturbed growth rates 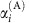 are determined such that the equilibrium abundances satisfy the fixed point equations obtained by equating to zero the right hand side of equation (1). We distinguish between facultative mutualism (the 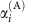 are positive) and obligatory mutualism (the 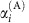 are negative, mutualistic interactions are necessary for surviance). We consider either mutualism that is facultative for both plants and animals or mutualism that is facultative for plants and obligatory for animals. Since the 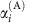 are not independent parameters, we must obtain obligatory mutualism by appropriately choosing the metaparameters.

We found that obligatory mutualism arises when the ratio between plant and animal biomasses 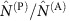 and the maximum mutualistic growth rates of animals 1/*H*^(A)^ are large. In fact, the feasibility of obligatory mutualism requires that the maximum mutualistic growth rate of animals, 1/*h*^(A)^, must be larger than the abundance loss at equilibrium because of competition,


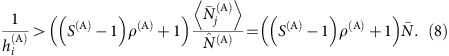


However, *h*_*i*_ cannot vanish, otherwise the equilibrium would be dynamically unstable^23^, and it also favours feasibility through the trade-off between the number and the strength of mutualistic interactions, since large numbers of mutualistic interactions approach saturation reducing their individual strength. Thus, for obligatory mutualism we choose *H*^(A)^ slightly smaller than the upper limit equation (8), *H*^(A)^=0.75/(*S*^(A)^*ρ*^(A)^+1−*ρ*^(A)^)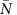. For plants there is no upper limit and we choose *H*^(P)^=0.25. Furthermore, the conditions that growth rates are negative for animals and positive for plants translate into the inequalities


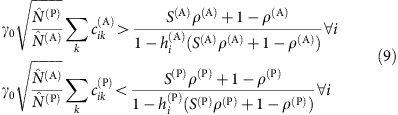


where we assumed that the equilibrium abundances are equal. Both conditions require that the ratio of the abundances between plant and animal populations 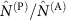 must be large. The most stringent is the first one obtained from the animals growth rates, which we write as


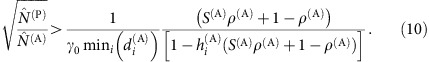


where 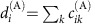 is the weighted degree of animal *i*. This condition requires that also equation (8) is fulfilled. If the smallest mutualistic degree is one, the number of animal species is *S*^(A)^=50, the competition is strong (*ρ*^(A)^=0.25) and the system is in the weak mutualist regime (*γ*_0_=0.1) we obtain 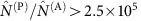. This value is consistent with empirical estimates^28^. Nevertheless, to achieve obligatory mutualism in a larger range of parameters, we use 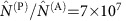.

### Parameter regimes

With our assumptions, the dynamical equations depend on the mutualistic network, on seven meta-parameters that determine the interaction matrices (*ρ*^(A)^, *ρ*^(P)^, *H*^(A)^, *H*^(P)^, *γ*_0_ and 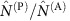), the equilibrium abundances 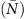 and through them the *α*_*i*_, and on the 3 parameters *δ*_*b*_, *δ*_*c*_ and *δ*_*N*_ that control the broadness of the distributions. We present results for several regimes of meta-parameters, described in Table 1.

### Dynamical stability

To assess dynamical stability, we take the derivatives of equation (1) close to the fixed point and we transform the dynamical system into an equivalent Lotka–Volterra (LV) system with effective mutualistic interactions 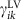 and effective growth rates 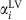 defined as


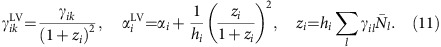


Any species can have either weak mutualism, if its equilibrium mutualistic growth rate is far from saturation 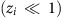, or strong mutualism 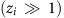.

The equilibrium is locally stable if the eigenvalues of the community matrix *J*_*ik*_=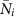
*A*_*ik*_ have negative real parts, where *A*_*ik*_ is the interaction matrix of the LV system. Furthermore, the LV system is globally stable if its interaction matrix is diagonally stable^20^, which also implies global stability of the complete equation (1)^21^. Both the local and global stability conditions require that the effective mutualistic interactions *γ*^LV^ are small enough. Since the *γ*^LV^ increase with *γ*_0_ in the weak regime and decrease in the strong regime, reaching a maximum in between, the equilibrium is stable both for *γ*_0_<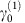 and for *γ*_0_>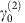. The saturation factors *z*_*i*_ are proportional to the products 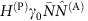 for plants and 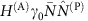 for animals. Clearly, increasing 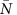 improves the stability.

For each set of meta-parameters and each network and for a typical realization of the random variables *b*_*ij*_, *c*_*ik*_ and *e*_*i*_, we numerically determine the critical mutualistic strengths 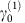 and 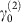 that guarantee local stability (see Supplementary Note 1 for details), and we use these computations to choose the values of *γ*_0_ adopted in our simulations.

### Numerical measure of structural stability

For each network and each set of metaparameters, we randomly draw 50 realizations of the interaction matrices and the equilibrium abundances, and we determine the critical values of *γ*_0_ at which the system looses dynamical stability (see above). Computations are only performed for *γ*_0_ in the allowed range for all networks. Subsequently, we generate 100 random perturbations of the intrinsic growth rates, 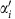
=*α*_*i*_(1+Δ*r*_*i*_), where *r*_*i*_ is a random number extracted in [−1, 1] and *α*_*i*_ are the unperturbed intrinsic growth rates. We integrate the ecological dynamics with the Bulirsch-Stoer algorithm with adaptive step until convergence, considering extinct species whose abundance falls below 10^−8^ of the initial value. For each Δ we record the fraction of simulations in which at least one species got extinct and we obtain through interpolation the critical perturbation Δ_c_ at which this fraction equals 0.5.

### Prediction of structural stability

We outline here the steps of the analytical prediction of structural stability, which are schematically represented in Fig. 3. The first step of the computation is the equivalent LV system used to compute dynamical stability, which represents the dynamics close to the fixed point. Second, we compute the effective competition matrix ***C***^(P)^=***β***^(P)^−***γ***^LV(P)^(***β***^(A)^)^−1^ ***γ***^LV(A)^ and the productivity vectors ***p***^(P)^=***α***^LV(P)^+***γ***^LV(P)^(***β***^(A)^)^−1^***α***^LV(A)^ and equivalent for animals^8^. Third, through diagonalization of these matrices we obtain the effective competition parameters *ρ*^eff(P)^ and *ρ*^eff(A)^,


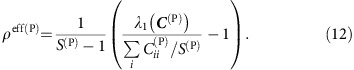


where *λ*_1_(***C***^(P)^) is the maximum eigenvalue of matrix ***C***^(P)^, together with the main eigenvectors ***v***^(P)1^ and equivalent for animals (see Supplementary Note 1 for the interpretation of these quantities). In the following, we omit the superscripts P or A whenever no ambiguity arises. It was shown in ref. 8 that the structural stability is inversely related with the effective competition parameter, in the sense that sufficient condition for all species having positive abundance larger than *n*_c_ (a threshold abundance below which a species is not viable) is that all productivities fulfil the inequalities


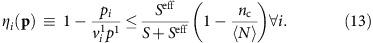


where *S*^eff^=(1−*ρ*^eff^)/*ρ*^eff^ is a biodiversity scale set by the effective competition. The larger *ρ*^eff^ or the number of species *S*, the more difficult is to satisfy the above conditions.

We call *η*_*i*_ the vulnerability factors. Interestingly, their weighted average is zero: 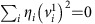. To compute how a perturbation of the intrinsic growth rates affects *η*_*i*_, we face the difficulty that the perturbation modifies the equilibrium abundances, and consequently the equivalent LV system. We simplify the computation by noting that, when mutualistic interactions are far or close to saturation, the changes in ***γ***^LV^ and ***α***^LV^ because of changes in equilibrium abundances are small and they can be neglected (except for obligatory mutualism, see below). Thus, we assume that the ***γ***^LV^, the effective competition matrices and their eigensystems are the same as in the unperturbed system. We extract perturbed growth rates 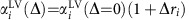, from them we compute the perturbed productivities *p*_*i*_(Δ), we project them onto the main eigenvector obtaining 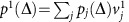 and the vulnerability factor 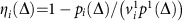. Finally, we compute *η*(Δ)=max_*i*_{*η*_*i*_(Δ)} and we average this quantity over 100 realizations of the perturbations. Each individual *η*_*i*_ is a linear function of Δ, however the most vulnerable species *v* is not the same at Δ=0 and at large Δ, so that *η*(Δ) is linear only for large enough Δ where *v* does not change, *η*(Δ)≈*η*_*v*_+Δ*η*′. Therefore, we obtain the propagation of perturbations and the unperturbed vulnerability as


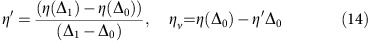


for Δ_0_=*η*_c_−0.05 and Δ_1_=*η*_c_+0.05.

For obligatory mutualism we have to consider that the plant abundance after the perturbation must remain large enough so that all 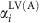 are positive. Thus, it is not justified to neglect the change in equilibrium abundances because of the perturbation. We consider the worst case of an animal species *i* that only feeds on a single plant *k*. Positivity of 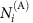 requires that


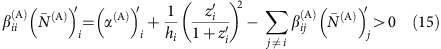


where 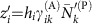 is the saturation factor after the perturbation. To focus on the saturation factor, we neglect changes of the term 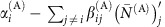, whose value before the perturbation is 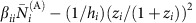, and we obtain the inequality


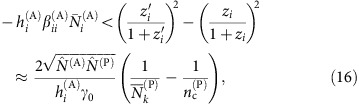


where 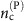 is the minimum plant abundance after the perturbation that can maintain the animal species, that is, the minimum abundance such that the above inequality holds, which we want to estimate, and we neglected terms of order 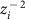 and 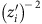. Multiplying both sides times *N*_*k*_^(P)^, using 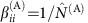 and approximating the abundances with the average over the community, 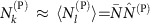 and 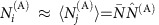, we can estimate the minimum plant abundance as


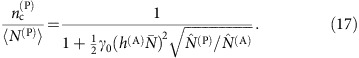


Equation (17) estimates the minimum abundance of plants in equation (13), while for animals we can consider 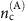
=0 since we set the unperturbed abundances such that 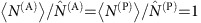. Using the fact that *η* is a linear function of Δ, *η*=*η*_*v*_+Δ*η*′, we find from equation (13)


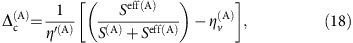



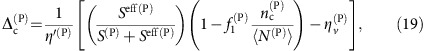


where 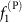 is the fraction of plant species that are the only connection of at least one animal species in obligatory mutualism. The quantity 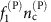/<*N*^(P)^> is indicated as 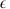 in the main text. Finally, 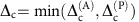.

### Predicting the propagation of perturbations

In equation (4), the propagation of perturbations *η*′ is numerically computed through eqution (14). We can predict it analytically in the same approximation used above. We consider perturbations *α*_*i*_→*α*_*i*_(1+Δ*r*_*i*_), where *r*_*i*_ are independent random variables with normal distribution. The effective growth rate is 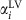
=*α*_*i*_+*m*_*i*_ and we neglect changes of *m*_*i*_=(1/*h*_*i*_)(*z*_*i*_/(1+*z*_*i*_))^2^ on perturbation. The productivity after perturbation is *p*_*i*_(Δ)=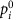
+Δ*q*_*i*_, where *q*_*i*_ is a Gaussian variable with mean zero and variance


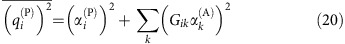


where *G*=*γ*^LV(P)^(*β*^(A)^)^−1^. To estimate *η*′, we compute *η*_*i*_(*p*(Δ)) at first order in Δ:


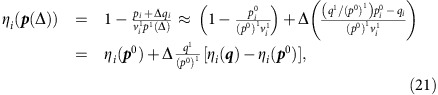


where we denote by *x*^1^ the projection of vector ***x*** on the main eigenvector ***v***^1^, 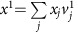. The resulting vulnerability *η*_*i*_ is a linear function of Δ that depends on the random variables *q*_*i*_ and *q*^1^. The variance of the denominator of equation (21) is given by 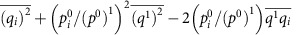. It is easy to see that 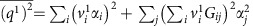 and 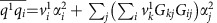. They both share the main qualitative property of 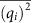, that is, they are all sums of *d*_*i*_+1 positive terms. Thus, to simplify formulas, in the following we estimate the variance of the denominator of equation (21) simply through 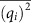.

The feasibility condition depends on the most vulnerable species, which is different in the unperturbed system Δ=0 (the species *i* that maximizes *η*_*i*_(***p***^0^)) and for large Δ (the species *v* that maximizes *η*_*i*_(Δ)). If Δ is large enough, the most vulnerable species is the species that maximizes *η*_*i*_(***q***)−*η*_*i*_(***p***^0^) and it does not change for larger Δ, so that the maximum value of *η*_*i*_(Δ) increases linearly as *η*(Δ)≡max_*i*_{*η*_*i*_(Δ)}≈*η*_*v*_(***p***^0^)+Δ*η*′(***p***^0^). *η*′ must be computed for the species with maximum *η*_*i*_, leading to


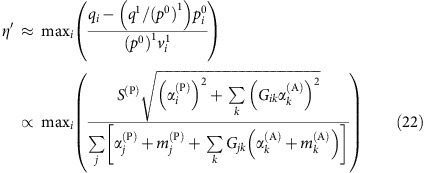


where we estimate *q*_*i*_ with its root mean square value multiplied times a constant, *v*_*i*_^1^≈1/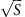 and 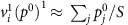, and we take into account that *p*^0^ depends on the effective growth rates 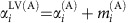.

The above formula is complex, and we prefer to compute *η*′ numerically with equation (14), but it makes clear two important qualitative points: (1) *η*_*i*_ decreases with the number of links (that is, the number of non-zero components *G*_*ik*_), 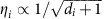, thus *η*′ decreases with the connectance of the mutualistic network and (2) *η*′ is larger for obligatory mutualism, in which the terms *α* and *m* have opposite sign.

### Analytical insights from effective competition

The effective competition matrix plays a central role in determining structural stability, dynamical stability and species abundances. This role stems from the equilibrium equations 
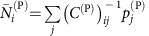
 and equivalent for animals. As it was shown in^8^, the structural stability is inversely related to the effective competition through equation (13). When *S* or *ρ*^eff^ are large, equation (13) implies that the productivity vector must be directed along ***v***^1^: 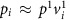. This in turn implies that the equilibrium abundances are also directed along ***v***^1^ and that they are also inversely related to *ρ*^eff^: 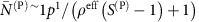. Finally, by computing the trace of the effective competition matrix we can easily see that *ρ*^eff^ is inversely related with the mean value of the minor eigenvalues of the effective competition matrix:


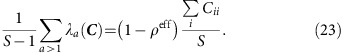


It was shown in ref. 21 that the diagonal positivity of the effective competition matrix is a necessary condition for global stability of the full dynamical system. This suggests that the larger the mean of the eigenvalues of the effective competition matrix is, the more likely it is that the system is globally stable, that is, we expect a negative relationship between *ρ*^eff^ and dynamical stability.

The central role of the effective competition matrix makes it useful to estimate its dependence on the network structure. To this end, we approximate the fully connected direct competition matrices with the mean-field matrices 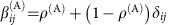 and equivalent for plants, we use rescaled units for simplicity, and we get


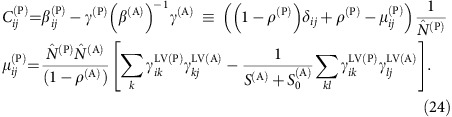


with *S*_0_^(A)^=(1−*ρ*^(A)^)/*ρ*^(A)^. Approximating 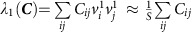, the effective interspecific competition equation (12) can be computed as


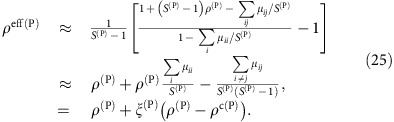


with 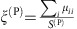, which justifies equation (2) in the main text. In other words, mutualistic interactions reduce the effective interspecific competition between plants, that is, *ρ*^eff(P)^<*ρ*^(P)^, only if the direct interspecific competition parameter *ρ*^(P)^ is smaller than the critical value *ρ*^c(P)^ given by


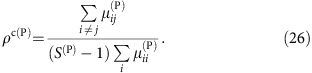


We can explicitly compute the matrix 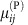 in three situations: when mutualistic interactions are far from saturation for all species (weak regime), close to saturation for all species (strong regime), or close to saturation for animals and far from plants (weak-strong regime), as in obligatory mutualism. For simplicity, in these computations we neglect the variability of the interaction coefficients , and instead of them we use the binary adjacency matrix *a*_*ik*_. We denote weak, strong and weak-strong regimes with the superscripts w, s and ws, respectively.

### Weak mutualism

If all mutualistic interactions are far from saturation 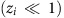 we approximate the effective mutualistic strengths as 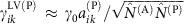. This is valid if all degrees 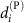 are smaller than the value 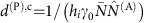, and equivalent for animals. In this case, it holds


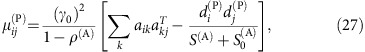


and a straightforward computation yields the critical competition and the coefficient


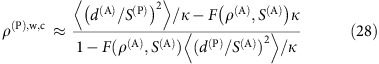



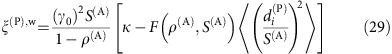


where 
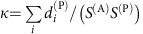
 is the connectance, 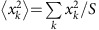 and


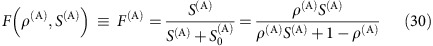


with *S*_0_^(A)^=(1−*ρ*^(A)^)/*ρ*^(A)^. *F*(*ρ*^(A)^, *S*^(A)^)≤1 is a measure of the richness of animal species with respect to the biodiversity scale 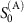. Since 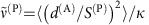 and 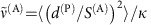 are well approximated by the nestedness of plants and animals, equation (5), we see that the critical competition increases with the nestedness, making it more likely that mutualism reduces the effective competition. For fully connected networks *a*_*ik*_≡1, *κ*=1, 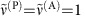 and *ρ*^c(P)^=1. Thus, we recover the result of^9^ that fully connected mutualistic networks always decrease the effective competition. The final result *ρ*^eff(P)^=*ρ*^(P)^−*γ*_0_^2^(1−*ρ*^(P)^)*F*^(A)^/*ρ*^(A)^ coincides for small *γ*_0_ with the formula reported in ref. 9.

### Strong mutualism

In the strong mutualism limit 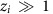 the effective mutualistic interactions are approximately given by 
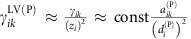
, with 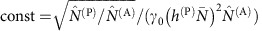. In this regime, the mutualistic matrix is given by


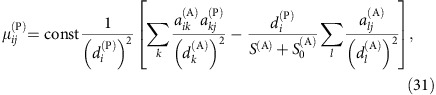


which yields the critical competition


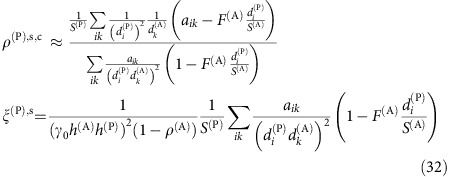


where *F*^(A)^ is given by equation (30). Once again, for fully connected networks it holds *ρ*^c(P)^=1, so that fully connected mutualistic networks decrease the effective competition. For sparse networks, the term at the denominator of equation (32) is always positive. For a random network with 
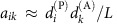
, we see that the term at the numerator is proportional to 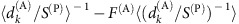, which is negative unless *κ*=1 or *F*^(A)^ is small. This suggests that in the strong mutualism regime it holds *ρ*^(P),s,c^<0 unless the connectance is large, and mutualistic interactions increase the effective interspecific competition for all values of *ρ*, as verified in our simulations.

### Obligatory (weak–strong) mutualism

Finally, in obligatory weak–strong mutualism animals are in the strong regime and plants are in the weak regime and it holds


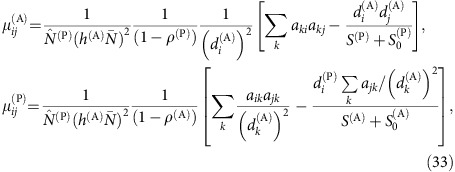


We see from this equation that the mutualistic matrix does not depend on *γ*_0_ for weak-strong mutualism, and therefore the effective competition parameter does not depend on *γ*_0_ either. The critical competition is


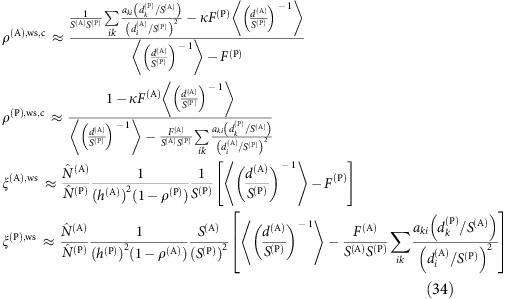


where 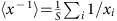. The deviation from pure competition is proportional to 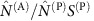, thus it is very small and it can be neglected.

### Data and software availability

The artificial mutualistic networks with different connectance and nestedness simulated in this study can be downloaded from the url http://ub.cbm.uam.es/research/TheoreticalEcology/TheoreticalEcology.php. The programs for the analytical prediction of structural stability given a mutualistic network and a set of metaparameters, and for the computation of the critical competition of a mutualistic network are available on request.

## Additional information

**How to cite this article:** Pascual-García, A. & Bastolla, U. Mutualism supports biodiversity when the direct competition is weak. *Nat. Commun.* **8,** 14326 doi: 10.1038/ncomms14326 (2017).

**Publisher’s note**: Springer Nature remains neutral with regard to jurisdictional claims in published maps and institutional affiliations.

## Supplementary Material

Supplementary Information Supplementary Figures, Supplementary Notes and Supplementary References

## Figures and Tables

**Table 1:**
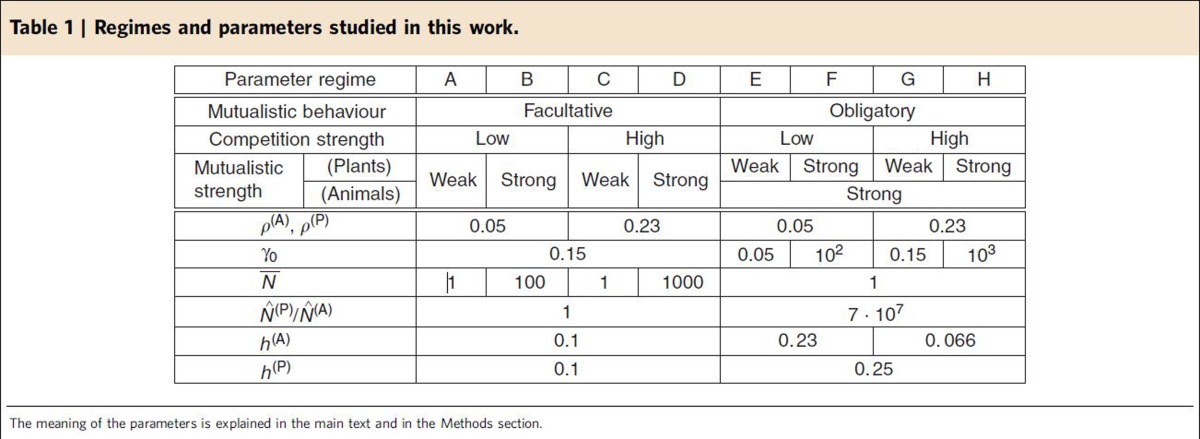
Regimes and parameters studied in this work.

The meaning of the parameters is explained in the main text and in the Methods section.
