# Supplementary material for: Mutualism supports biodiversity when the direct competition is weak
Source: Nat Commun. 2017 Feb 24;8:14326. doi: 10.1038/ncomms14326 (PMC5512850; doi:10.1038/ncomms14326)
Supplement: Supplementary Information — Supplementary Figures, Supplementary Notes and Supplementary References [file ncomms14326-s1.pdf]

Supplementary Note 1:
Ecosystem stability and the effective competition framework

Dynamical system

As in Refs. [1–3], the multi-species population dynamics is governed by the ordinary differential equations

$$\frac{1}{N_i^{(\mathrm{P})}}\frac{\mathrm{d}N_i^{(\mathrm{P})}}{\mathrm{d}t} = \alpha_i^{(\mathrm{P})} - \sum_{j\in\mathbf{P}} \beta_{ij}^{(\mathrm{P})} N_j^{(\mathrm{P})} + \sum_{k\in\mathbf{A}} \frac{\gamma_{ik}^{(\mathrm{P})} N_k^{(\mathrm{A})}}{1 + h_i^{(\mathrm{P})} \sum_{l\in\mathbf{A}} \gamma_{il}^{(\mathrm{P})} N_l^{(\mathrm{A})}} \,. \tag{1}$$

where $N_i^{(\mathrm{P})}$ denotes the abundance of plant species i, $\alpha_i^{(\mathrm{P})}$ is its intrinsic growth rate in the absence of other species, $\beta_{ij}^{(\mathrm{P})}$ is the direct competition matrix and $\gamma_{ik}^{(\mathrm{P})}$ is the mutualistic matrix. The equations and parameters for animals are obtained interchanging the superscripts P and A and they will be presented only when necessary. We will sometimes use the superscript X to indicate either group.

We adopt the so-called soft mean field model, which assumes that all equivalent interaction parameters are identically distributed. The competition network is fully connected, while the mutualistic network is sparsely connected, with adjacency matrix a_{ik}, and its interactions are parameterized as $\gamma_{ik}^{(\mathrm{P})} = a_{ik}\gamma_0 g_{ik}^{(\mathrm{P})}$. The parameter γ_0, which represents the strength of the mutualistic interactions with respect to competitive ones, is chosen in a range such that the dynamical system is globally stable for all the studied mutualistic networks (see subsection Equivalent Lotka-Volterra system and dynamical stability).

Given a realization of the ecological interactions β_{ij} and γ_{ik}, if we randomly extract the intrinsic growth rates α_i we obtain with high probability equilibrium abundances that are not positive (i.e. the equilibrium is not feasible), in particular if the degrees of the mutualistic network are broadly distributed and we extract the α_i independent of these degrees. Therefore, instead of randomly extracting the intrinsic growth rates, we impose that the equilibrium is feasible and dynamically stable and study how structurally stable it is against random perturbations of the growth rates.

The first step in our numerical experiments consists in choosing positive equilibrium abundances $\overline{N}_i > 0$ for all species, i.e. we guarantee the feasibility of the equilibrium. We then determine the unperturbed growth rates $\overline{\alpha_i^{(\mathrm{A})}}$ in such a way that these $\overline{N}_i$ are a fixed point of the dynamical system:

$$\overline{\alpha_i^{(\mathrm{P})}} = \sum_{j\in\mathbf{P}} \beta_{ij}^{(\mathrm{P})} \overline{N}_j^{(\mathrm{P})} - \sum_{k\in\mathbf{A}} \frac{\gamma_{ik}^{(\mathrm{P})} \overline{N}_k^{(\mathrm{A})}}{1 + h_i^{(\mathrm{P})} \sum_{l\in\mathbf{A}} \gamma_{il}^{(\mathrm{P})} \overline{N}_l^{(\mathrm{A})}} \,. \tag{2}$$

Note that, if the abundances are narrowly distributed, the unperturbed growth rates are negatively correlated with the number of non-zero mutualistic interactions γ_{ik}, which can be interpreted as a trade-off between the number of interactions and their metabolic cost.

Equivalent Lotka-Volterra system and dynamical stability

Close to a dynamical equilibrium, the dynamical stability for small perturbations of the abundances is determined by the equivalent Lotka-Volterra (LV) system

$$\frac{1}{N_i^{(\mathrm{P})}} \frac{\mathrm{d}N_i^{(\mathrm{P})}}{\mathrm{d}t} = \alpha_i^{\mathrm{LV(P)}} - \sum_{j \in \mathbf{P}} \beta_{ij}^{(\mathrm{P})} N_j^{(\mathrm{P})} + \sum_{k \in \mathbf{A}} \gamma_{ik}^{\mathrm{LV(P)}} N_k^{(\mathrm{A})} . \tag{3}$$

The effective interaction and growth rates parameters are obtained by differentiating the full dynamical equations Supp.Eq.(1) at the equilibrium point, and they are

$$\gamma_{ik}^{\mathrm{LV(P)}} = \frac{\gamma_{ik}^{(\mathrm{P})}}{(1+z_i)^2} \tag{4}$$

$$\alpha_i^{\mathrm{LV(P)}} = \alpha_i^{(\mathrm{P})} + \frac{1}{h_i^{(\mathrm{P})}} \left(\frac{z_i}{1+z_i} \right)^2 . \tag{5}$$

$$z_i = h_i^{(\mathrm{P})} \sum_{l \in \mathbf{A}} \gamma_{il}^{(\mathrm{P})} \overline{N}_l^{(\mathrm{A})}$$

We see from this equation that for each species there are two regimes of parameters: weak mutualism, in which the equilibrium mutualistic benefit is far from saturation ($z_i \ll 1$) and strong mutualism, in which the saturation is reached ($z_i \gg 1$). Thus, it is possible that, for given meta-parameters, species with large degree are in the strong mutualistic regime while species with small degree are in the weak regime. The effective mutualistic strength increases with γ_0 in the weak mutualistic regime and decreases in the strong regime, reaching a maximum in between.

The local stability of the equilibrium point can be tested from the linearized system Supp.Eq.(3), which we rewrite as $\frac{1}{N_i}\frac{\mathrm{d}N_i}{\mathrm{d}t} = \alpha_i - \sum_j A_{ij} N_j$. The equilibrium is locally stable if and only if all eigenvalues of the Jacobian matrix $J_{ik} = -\overline{N}_i A_{ik}$ have negative real part. This requires that the elements of the matrices γ^{LV} are small. From Supp.Eq.(4), it is clear that this happens both for small and for large values of γ_0, i.e. the equilibrium is stable if $\gamma_0 < \gamma_0^{(1)}$ or $\gamma_0 > \gamma_0^{(2)}$, which define the lower and upper critical mutualistic strengths and the weak and strong mutualistic regimes, respectively. We determine numerically the critical strengths for each given network and given realization of the interaction parameters by diagonalizing the matrix J_{ik}.

We can obtain greater analytic insight on dynamical stability using the effective competition matrix defined below.

The effective competition framework

The effective competition matrix represents the interactions between species in the same group, either P or A, both due to their direct interaction (in this case, competition) and to their interaction with species in the other group (in this case mutualism). It allows to decouple the fixed point equations as

$$\overline{N}^{(\mathrm{P})} = \left(C^{(\mathrm{P})}\right)^{-1} p^{(\mathrm{P})} \qquad \overline{N}^{(\mathrm{A})} = \left(C^{(\mathrm{A})}\right)^{-1} p^{(\mathrm{A})} \tag{6}$$

where the effective competition matrices C and the effective productivity vectors p are defined as

$$C^{(A)} = \beta^{(A)} - \gamma^{LV(A)} \left(\beta^{(P)}\right)^{-1} \gamma^{LV(P)}, \qquad p^{(A)} = \alpha^{LV(A)} + \gamma^{LV(A)} \left(\beta^{(P)}\right)^{-1} \alpha^{LV(P)} \quad (7)$$

and analogous for plants [4].

The usefulness of the effective competition matrix stems from the fact that, while the full interaction matrix A has both positive and negative components, we expect that most elements of the matrix $C^{(P)}$ and $C^{(A)}$ are positive in the regime in which the system is dynamically stable (for simplicity, we omit here the superscripts since the properties that we describe are common to both matrices). In particular, we assume that the components of C are non-negative and that the matrix is irreducible, i.e. the complete space is the only spaces that is invariant under the action of C, so that we can apply the Perron-Frobenius theorem [5] that states that each matrix C has a dominant eigenvalue λ_1 of order S (the dimension of the space) associated with left and right eigenvectors u^1 and v^1 whose elements are all positive, while all other eigenvectors have at least one negative or complex component.

The main eigenvalue λ_1 can be interpreted as the weighted average of the matrix C with weights given by the main left and right eigenvectors, $\lambda_1 = \sum_{ij} C_{ij} u_i^1 u_j^1 = \sum_{ij} C_{ij} v_i^1 v_j^1$. As shown in [4], it is convenient to change abundance units in such a way that the intraspecific competition is equal to one, i.e. $\tilde{N}_i = N_i \sqrt{C_{ii}}$, $\tilde{C}_{ij} = \frac{C_{ij}}{\sqrt{C_{ii} C_{jj}}}$ and $\tilde{p}_i = \frac{p_i}{\sqrt{C_{ii}}}$. This transformation preserves the fixed point equations, Supp.Eq.(6). From the main eigenvalue of the transformed effective competition matrix we can compute the effective interspecific competition parameter as [4]

$$\rho^{\text{eff}} = \frac{\lambda_1(\tilde{C}) - 1}{S - 1}. \quad (8)$$

Since $\tilde{C}_{ii} = 1$, we can write $\rho^{\text{eff}} = \sum_{i \neq j} C_{ij} v_i^1 v_j^1 / (S-1)$, which shows that ρ^{eff} is proportional to the weighted average of the interspecific competition, justifying its name. Since the trace of the matrix $\sum_i \tilde{C}_{ii} = S$ is equal to the sum of the eigenvalues, $1 - \rho^{\text{eff}}$ represents the average value of the minor eigenvalues: $\sum_{\alpha > 1} \lambda_\alpha / (S-1) = 1 - \rho^{\text{eff}}$. This is the key property that we used for deriving Supp.Eq.(11), based on which we predict structural stability (see below).

Here we adopted an alternative and more general way of determining the parameter ρ^{eff} that does not require rescaling the competition matrix. We have found that this procedure yields slightly more accurate predictions of the structural stability. The formulas reported above are recovered as a special case when $C_{ii} = 1$. We define ρ^{eff} as the ratio between interspecific and intraspecific competition,

$$\rho^{\text{eff}} = \frac{1}{S-1} \left(\frac{\lambda_1(C)}{\sum_i C_{ii}/S} - 1 \right). \quad (9)$$

Computing the trace, we recover the result that $1 - \rho^{\text{eff}}$ is proportional to the average value of the minor eigenvalues,

$$\frac{1}{S-1} \sum_{\alpha > 1} \lambda_\alpha(C) = \left(1 - \rho^{\text{eff}}\right) \frac{\sum_i C_{ii}}{S}. \quad (10)$$

However, note that with the above definition ρ^{eff} is not anymore proportional to the weighted average of the interspecific competition.

The effective competition parameter ρ^{eff} plays a central role in determining the dynamical stability, structural stability and abundance of the model ecosystem, as shown in previous works [4, 6, 7] and recalled below for completeness.

Types of stability

The stability of the dynamical system Supp.Eq.(1) can be defined in several ways. Dynamical stability refers to perturbations of the dynamical variables N_i, and it can be can be local or global. Local stability against small perturbations of the dynamical variables around their equilibrium values is analytically studied considering the linearized dynamical system Supp.Eq.(3). Global stability refers to perturbations of whatever size of the dynamical variables. Goh has shown that, if an interaction matrix A is diagonally positive, meaning that there is a diagonal and positive matrix D such that the symmetric matrix $DA + A^T D$ is positive definite, then the Lotka-Volterra system defined by this matrix, $\frac{1}{N_i}\frac{\mathrm{d}N_i}{\mathrm{d}t} = \alpha_i - \sum_j A_{ij}N_j$ is globally stable provided that the equilibrium is feasible, $\overline{N}_i > 0\ \forall i$ [8][1]. This implies that, for whatever perturbation of the dynamical variable, no species will get extinct provided that the parameters of the system allow feasibility. In this situation, it is possible to analytically investigate a more general type of stability, namely structural stability.

A dynamical system is said to be structurally stable if its qualitative properties do not change when its parameters suffer a perturbation. If the interaction matrix is diagonally stable, then structural stability against perturbations of the growth rates can be defined as the maximum perturbation that maintains a feasible equilibrium in which all species coexist. Since the interaction matrix does not change, its global stability remains guaranteed. We can define in an analogous way the structural stability with respect to changes in the interaction matrix, but in this case we have also to test that the matrix remains diagonally positive.

Effective competition and dynamical stability

The dynamical stability of LV systems is usually addressed in terms of the complete interaction matrix A, which involves both groups of plants and animals. Recently, we have addressed dynamical stability through the effective competition matrix of each group of species separately [6]. For this purpose, we used Goh's theorem mentioned above [8] that states that a LV system is globally stable if its interaction matrix A is diagonally positive (with the definition of A that we adopted; with the conventional definition it must be diagonally negative, see previous section).

In particular, we have shown that diagonal positivity of the effective competition matrix $C^{(\mathrm{P})}$ and the conjugated direct competition matrix $\beta^{(\mathrm{A})}$ is a necessary condition for diagonal positivity of the complete interaction matrix A, which in turn is sufficient for

[1] Note that usually the interaction matrix A is defined with the opposite sign and the sufficient condition of global stability is diagonal negativity of the matrix A. We define the matrix A with the changed sign in order to make the correspondence with the effective competition matrix easier.

global stability. If A is symmetric, then the above condition is necessary and sufficient for diagonal positivity of A. If it is not, mild symmetry conditions on the main principal vectors of the intergroup interaction matrices must be also imposed to guarantee the diagonal positivity of A [6]. Furthermore, we have shown in [6] that diagonal positivity of the LV interaction matrix A is also a sufficient condition for global stability of the dynamical equations Supp.Eq.(1) with their saturation terms.

Diagonal positivity is not simple to test numerically, because we must find a suitable matrix D or rule out its existence. However, for the model that we simulated the interaction matrix is almost symmetric, since the direct competition matrices are approximately symmetric and the mutualistic interaction coefficients are the same for plants and animals. Therefore, we conjecture that for our model positivity of $C^{(\mathrm{P})}$ and $\beta^{(\mathrm{A})}$ is an almost necessary and sufficient for global stability of the linearized system, which implies that no species will get extinct provided that the equilibrium is feasible, as Supp.Eq.(2) guarantees by construction. Since also $\beta^{(\mathrm{A})}$ is positive definite and almost symmetric by construction, it is enough to test that $C^{(\mathrm{P})}$ is positive definite.

We tested numerically that the threshold value of γ_0 obtained through the condition that $C^{(\mathrm{P})}$ is positive definite, and which is almost sufficient for global stability, approximately coincides with the threshold value obtained from the local stability condition that $\mathrm{Re}\left(\lambda\left(J_{ik}\right)\right) < 0$ (see Supp.Fig.1). In all cases that we investigated numerically, local stability also guaranteed global stability.

Because of Supp.Eq.(10), the smaller is $\rho^{\mathrm{eff(P)}}$, the larger is the average of the minor eigenvalues of $C^{(\mathrm{P})}$ and the less likely it is that the minimum eigenvalue of $C^{(\mathrm{P})}$ is negative and the system is dynamically unstable. Therefore, we expect that dynamical stability is inversely related with $\rho^{\mathrm{eff(P)}}$, in particular that the lower mutualistic threshold $\gamma_0^{(1)}$ is inversely related with $\rho^{\mathrm{eff(P)}}$, consistent with our numerical results reported in Supp.Fig.2.

This relationship also predicts that the critical mutualistic strengths are influenced by the same network properties that influence $\rho^{\mathrm{eff(P)}}$, i.e. nestedness in the weak mutualistic regime (Supp.Fig.3 left plot) and connectance in the strong mutualistic regime (Supp.Fig.3 right plot).

Effective competition and structural stability

In this section, to simplify the notation we omit the superscripts P and A whenever no ambiguity arises. The structural stability with respect to changes in the productivities is in large extent determined by the effective interspecific competition ρ^{eff} [4, 7]. Sufficient condition for all species having positive abundance larger than n_{c} (a threshold abundance below which a species is not viable) is that all productivities fulfill the inequality

$$\eta_i(p) \equiv 1 - \frac{p_i}{v_i^1 p^1} \leq \frac{S^{\mathrm{eff}}}{S + S^{\mathrm{eff}}} \left(1 - \frac{n_{\mathrm{c}}}{\langle N \rangle}\right) \forall i \,. \tag{11}$$

We call η_i the vulnerability factor. Here v^1 is the main eigenvector of the effective competition matrix C, $p^1 = \sum_i p_i v_i^1$ is the projection of the productivity vector onto v^1, and S^{eff} is a natural biodiversity scale set by the effective competition,

$$S^{\mathrm{eff}} = \frac{1 - \rho^{\mathrm{eff}}}{\rho^{\mathrm{eff}}} \,. \tag{12}$$

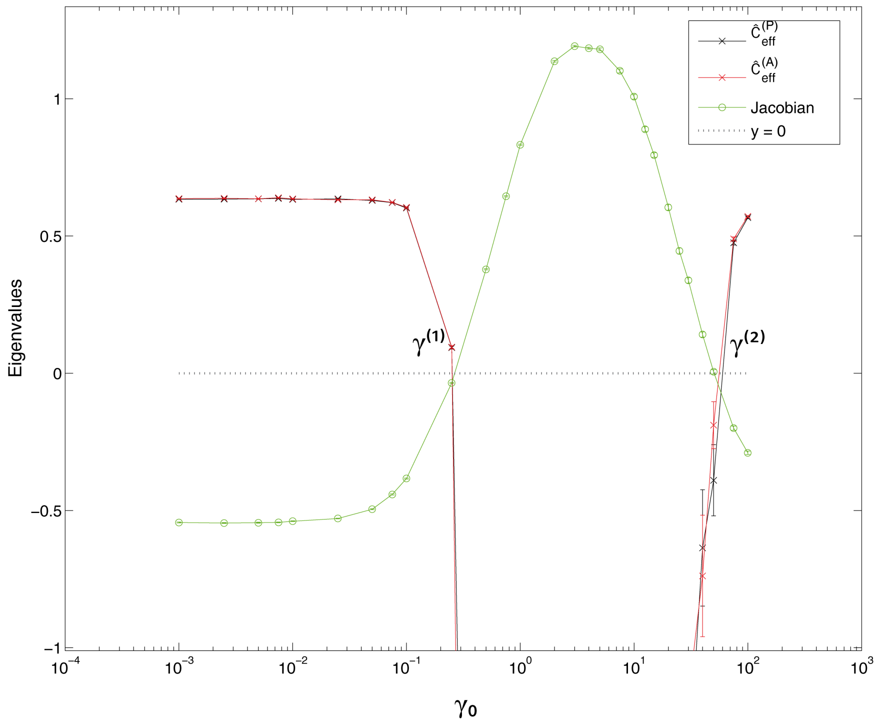

Supplementary Figure 1: **Local stability.** Maximum eigenvalue of the Jacobian matrix of the dynamical equations at the equilibrium point (green line) and minimum eigenvalues of the effective competition matrices for plants (black) and animals (red) as a function of the mutualistic strength γ_0. The system is dynamically unstable when the eigenvalue of the Jacobian is positive. Each point represents the average over 50 realizations of the interaction parameters b_{ij}, c_{ij} and e_i for one particular network with $S^{(P)} = 46$, $S^{(A)} = 47$, connectance 0.073 and nestedness 0.149, $\hat{N}^{(A)}/\hat{N}^{(P)} = 1$, $\rho^{(P)} = \rho^{(A)} = 0.23$.

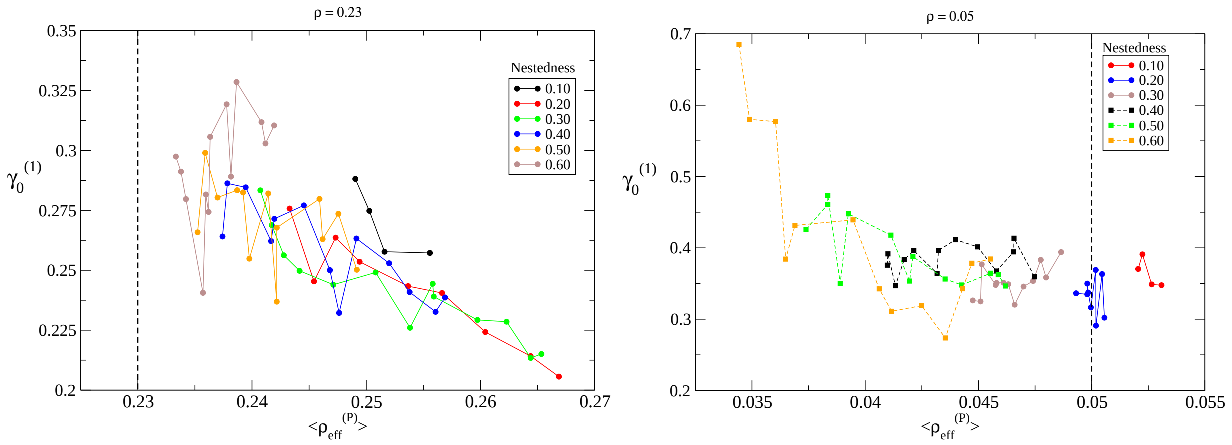

Supplementary Figure 2: **Critical mutualistic strength $\gamma_0^{(1)}$ versus the effective competition parameter $\rho^{\rm eff}$ for facultative mutualism.** Each point represents one network with different connectance and nestedness, and for each of them we performed 50 independent simulations. The dashed vertical line represents the effective competition in the absence of mutualistic interactions. Left: strong direct competition, $\rho = 0.23$. Right: weak direct competition, $\rho = 0.05$. Note that $\gamma_0^{(1)}$ and $\rho^{\rm eff}$ are inversely related in both cases.

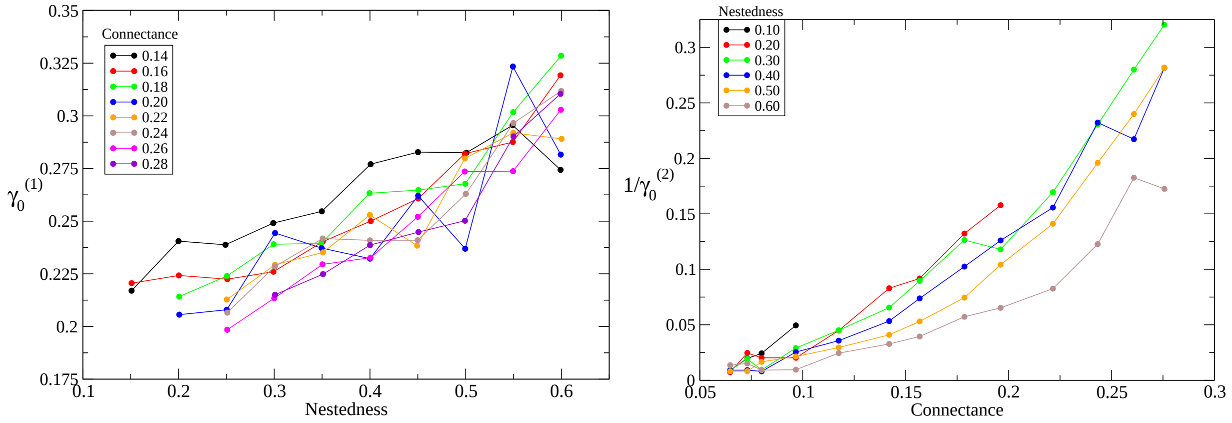

Supplementary Figure 3: **Critical mutualistic strengths versus network properties.** Each point represents one network with different connectance and nestedness, and for each of them we performed 50 independent simulations. Left: Lower critical strength $\gamma_0^{(1)}$ versus nestedness for large connectance. $\gamma_0^{(1)}$ depends little on nestedness for small connectance (not shown). Right: inverse of the upper critical strength $1/\gamma_0^{(2)}$ versus connectance. Data are for facultative mutualism with $\overline{N} = 1$.

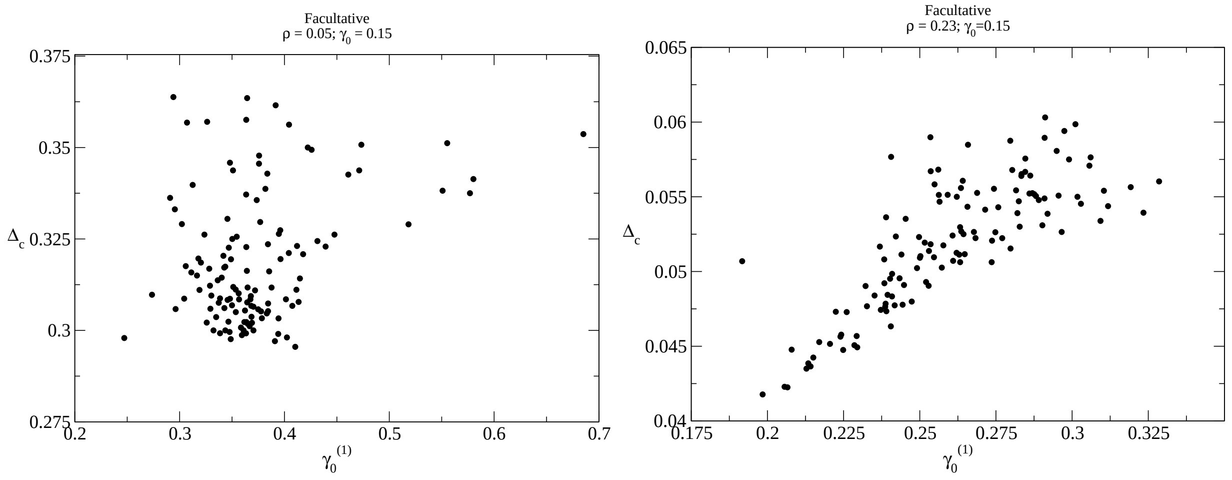

Supplementary Figure 4: **Structural stability Δ_c versus dynamical stability $\gamma_0^{(1)}$ for facultative weak mutualism.** Each point represents one network with different connectance and nestedness, and for each of them we performed 50 independent simulations. Left: weak competition $\rho = 0.05$. Right: strong competition $\rho = 0.23$. Each point represents a network with different connectance and nestedness.

If S^{eff}/S is small (either large ρ^{eff} or large S) all of the η_i must be close to zero (note that the weighted average of $\eta_i(p)$ is $\sum_i \eta_i(p)(v_i^1)^2 = 0$ for all vectors p), i.e. the productivity vector must be almost parallel to v^1, so that the feasibility condition Supp.Eq.(11) is very demanding and even small perturbations of the productivities can violate it.

The feasibility condition Supp.Eq.(11) is a sufficient condition. Nevertheless, we have observed with numerical experiments that it becomes an approximately necesary condition for feasibility when the productivity is exposed to random perturbations [7], and we will use it for predicting the critical perturbation Δ_c.

Structural stability and dynamical stability are positively related

As a corollary of the above results, in the regime A in which Δ_c is negatively influenced by ρ^{eff} we expect a negative correlation between the dynamical stability with respect to fluctuations of the species abundances, measured through $\gamma_0^{(1)}$, and the structural stability with respect to fluctuations of growth rates, measured through Δ_c. Conversely, in the regimes in which Δ_c is mainly influenced by the propagation of perturbations, both Δ_c and $\gamma_0^{(1)}$ increase with the connectance of the mutualistic network and we expect that they are positively related. This implies that the structural stability with respect to variation in the mutualistic interactions and with respect to variations in the intrinsic growth rates go in the same direction in these regimes. This prediction is supported by our numerical results reported in Supp.Fig.4.

Effective competition and equilibrium abundances

In the limit of vanishing S^{eff}/S, the feasibility condition implies that the productivity vector must be directed along v^1, $p_i \approx p^1 v_i^1$. Through the equilibrium equation $N =$

$(C)^{-1}\, p$, this in turn implies that the equilibrium abundances are also directed along v^1:

$$\overline{N}_i^{(\mathrm{P})} = \frac{v_i^1 p^1}{\rho^{\mathrm{eff}}(S^{(\mathrm{P})}-1)+1} \frac{S}{\sum_i C_{ii}}\,. \tag{13}$$

Thus for large systems with large S/S^{eff} whose productivities are strongly constrained, the equilibrium abundances are expected to be inversely related with ρ^{eff}, with the consequence that a decrease of ρ^{eff} positively affects structural stability, dynamical stability and equilibrium abundances.

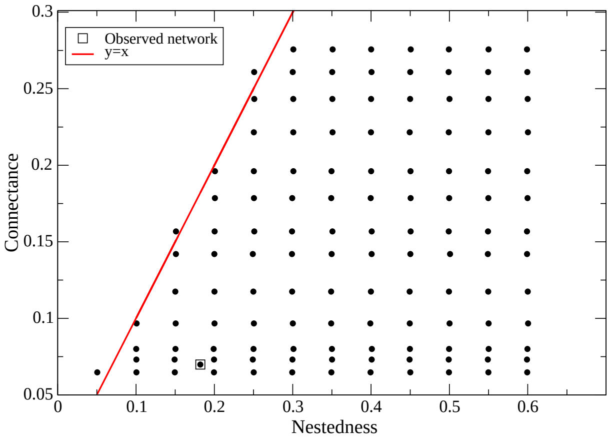

Supplementary Figure 5: **Nestedness and connectance for the pool of simulated networks.** The number of species is $S^{(\mathrm{P})} = 46$, $S^{(\mathrm{A})} = 47$. The observed network from which the simulated networks are derived has connectance 0.073 and nestedness 0.149.

Supplementary Note 2: Supplementary numerical results

Connectance and nestedness of the random networks

For each set of meta-parameters we considered 125 mutualistic networks with different combinations of connectance and nestedness. The properties of the simulated networks are depicted in Supp.Fig.5, where one can see that almost all networks have nestedness larger than connectance, a limit that is very difficult to overcome with the Monte Carlo schemes that we implemented.

Regimes not shown in the main text

We show in Supp.Fig.6 how structural stability Δ_{c}, effective interspecific competition ρ^{eff} and propagation of perturbations η' depend on network properties for some regimes of parameters that are not represented in the main text.

Supplementary Figure 6: **Structural stability and related quantities versus network architecture** (next page). Δ_{c} quantitfies structural stability, ρ^{eff} quantifies the effective competition and η' quantifies the propagation of perturbations. Each panel depicts a different regime versus connectance (left column) and nestedness (right column). Top panels: facultative mutualism, large equilibrium abundance, strong (left) and weak (right) direct competition. Intermediate panels: obligatory mutualism, weak mutualistic strengths, strong (left) and weak (right) direct competition. Bottom panels: obligatory mutualism, large mutualistic strength, strong (left) and weak (right) direct competition. Error bars represent the standard error of the mean over 50 realizations of the parameters and the ecological dynamics. The horizontal lines refers to absence of inter-species interactions, i.e. pure direct competition.

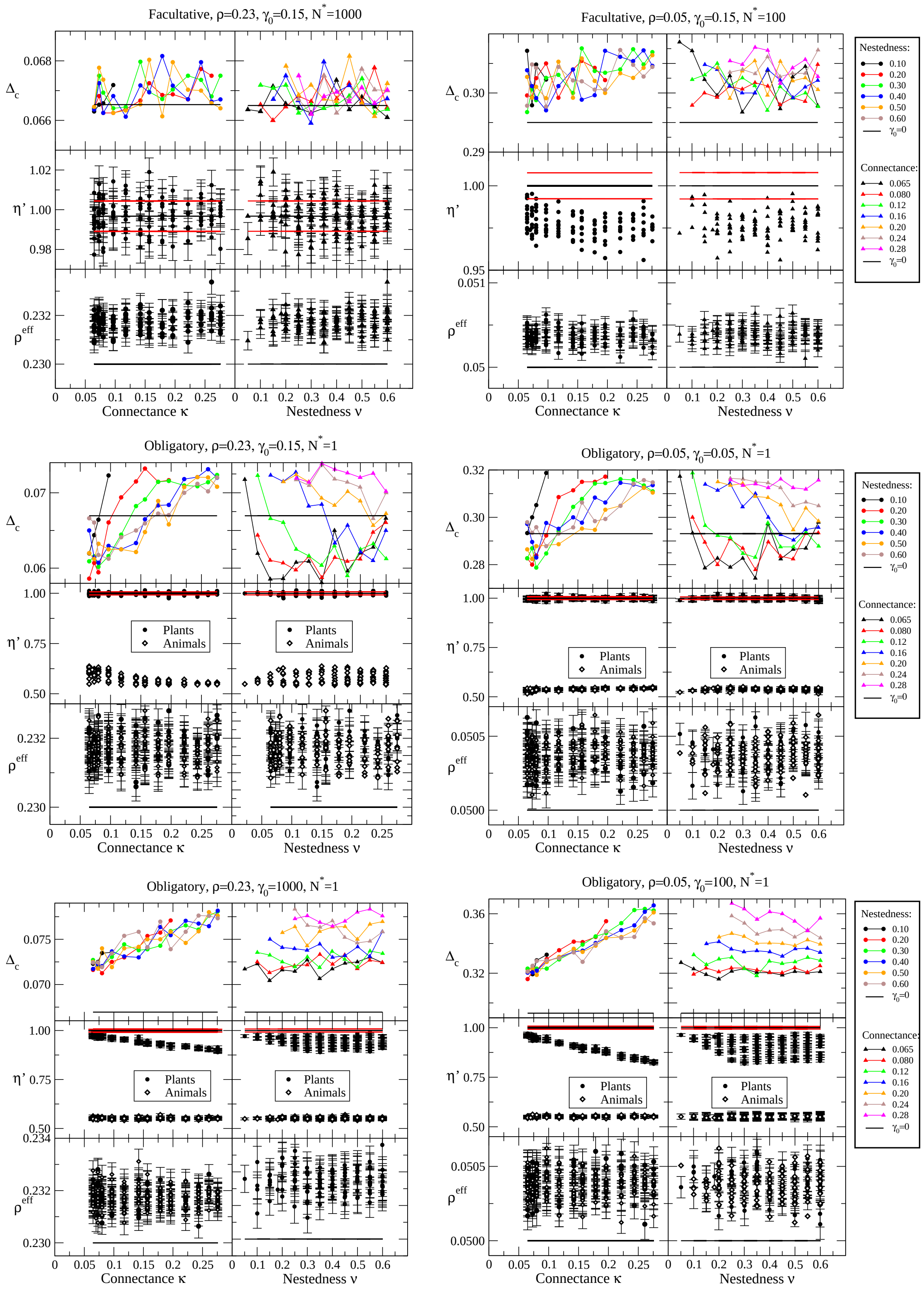

Facultative, ρ=0.23, γ0=0.15, N*=1000
Facultative, ρ=0.05, γ0=0.15, N*=100
Obligatory, ρ=0.23, γ0=0.15, N*=1
Obligatory, ρ=0.05, γ0=0.05, N*=1
Obligatory, ρ=0.23, γ0=1000, N*=1
Obligatory, ρ=0.05, γ0=100, N*=1
Δc
η'
ρeff
Connectance κ
Nestedness ν
Plants
Animals
Nestedness:
0.10
0.20
0.30
0.40
0.50
0.60
γ0=0
Connectance:
0.065
0.080
0.12
0.16
0.20
0.24
0.28
γ0=0

Effective competition parameter versus nestedness

We show in Supp.Fig.7 the effective competition parameter ρ^{eff} versus nestedness for different values of γ_0 and ρ. In the weak mutualism regime ρ^{eff} decreases with nestedness, in agreement with the results of the previous section. The critical competition increases with nestedness, and it is given by the point where $\rho^{\text{eff}} = \rho$.

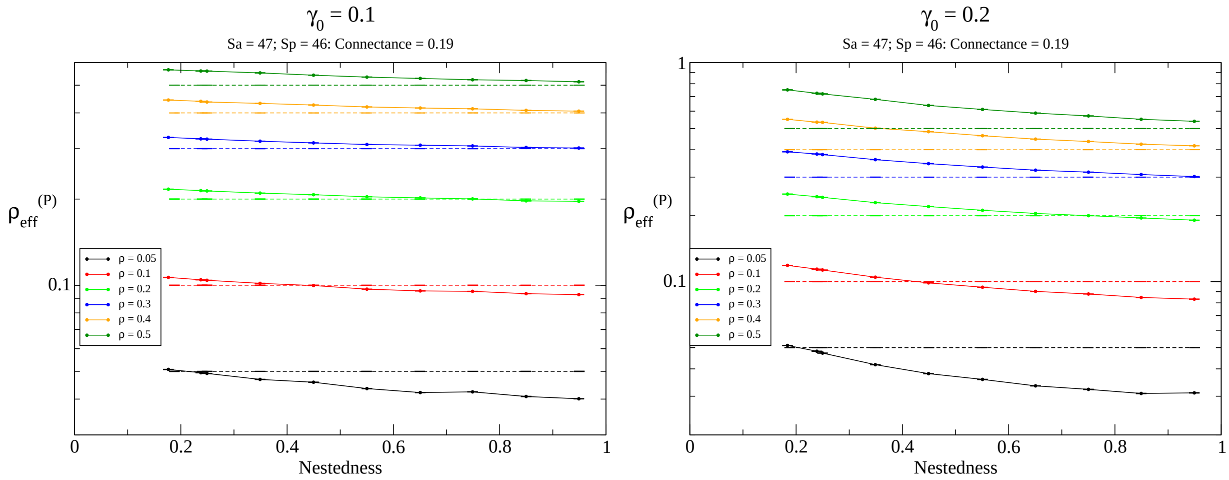

Supplementary Figure 7: **Interspecific effective competition parameter ρ^{eff} versus nestedness.** In all cases the connectance is $\kappa = 0.19$. Left: $\gamma_0 = 0.1$. Right: $\gamma_0 = 0.2$. Each pair of curves of the same colour refers to a different value of the direct competition parameter ρ. The dashed curve is for pure competition and the solid curve for mutualism. Note that the critical interspecific competition at which $\rho^{\text{eff}} = \rho$ depends on the nestedness but not on γ_0, which only influences the absolute value of the difference $\rho^{\text{eff}} - \rho$. Error bars represent the standard error of the mean over 50 realizations of the parameters and the ecological dynamics.

Supplementary Note 3: Influence of the unperturbed abundance distribution on structural stability

As we explained in the Methods section, we extract the unperturbed equilibrium abundances from distributions with mean value one and coefficient of variation CV (standard deviation divided by mean). We have presented in the main text results obtained with an unperturbed system with a narrow abundance distribution. Unperturbed abundances are drawn from an uniform distribution in $[0.85, 1.15]$ and have small coefficient of variation, i.e. ratio between standard deviation and mean, CV=0.087. This is in line with our previous study [3] and with Ref. [9], where the unperturbed system was constructed such that $p^0 \propto v^1$, from which we expect that the vulnerability of the unperturbed system is $\eta_v(p^0) \approx 0$, corresponding to a narrow distribution of equilibrium abundances (see below). The assumption that the distribution of abundances is narrow is justified by the results that we report in this Note, where we find that structural stability tends to vanish when the distribution of the unperturbed abundances approaches a critical value.
To put this result in context, we have to consider that here we adopt species-specific units such that the intraspecific competition coefficients β_{ii}, inversely related with the carrying capacities, are equal to one for all species of animals or plants. Starting from the original units of abundance, for instance number of individuals per square kilometre, we obtain a mathematically equivalent system of equations with $\tilde{\beta}_{ii} \equiv 1$ through the change of units $\tilde{N}_i = N_i\sqrt{\beta_{ii}}$, $\tilde{\beta}_{ij} = \beta_{ij}/\sqrt{\beta_{ii}\beta_{jj}}$, $\tilde{\alpha}_i = \alpha_i/\sqrt{\beta_{ii}}$. Thus, it is possible that the abundances N_i are broadly distributed in the original units, but they are narrowly distributed in the rescaled units $\tilde{N}_i = N_i\sqrt{\beta_{ii}}$ that enforce $\tilde{\beta}_{ii} = 1$. We can restate the result presented in this Note by stating that, if the distribution of abundances is large when measured in species-specific units such that $\tilde{\beta}_{ii} \equiv 1$, then the vulnerability of the unperturbed system is large and its structural stability is very small.
In this Note, we present the analytic computation of the influence of the unperturbed system on structural stability, tested with simulations performed with equilibrium abundances with lognormal distributions with large CV equal to 0.15, 0.30 and 0.95. These numerical experiments were performed in the weak facultative mutualism regime ($\gamma_0 = 0.15 < \gamma_0^{(1)}, \overline{N} = 1$), but the analytic predictions are valid for all regimes.

Effective abundances and structural stability

For completeness, we recall here the computation that shows how the unperturbed abundances influence structural stability. Again we omit the superscripts P and A unless there is some ambiguity.
The structural stability of the system with respect to changes in the intrinsic growth rates α_i depends on how these changes propagate onto the productivities and, through them, onto changes of the vulnerability factors η_i, Supp.Eq.(11). This computation is complicated by the fact that a change in α_i modifies the equilibrium abundances, and consequently the saturation factors z_i and, through them, the effective growth rates and mutualistic interactions, Supp.Eq.(5) and Supp.Eq.(4). Therefore, we would need to

explicitly compute the perturbed abundances and z_i, which would make the feasibility condition Supp.Eq.(11) useless.
Nevertheless, we can get analytic insight by noticing that, when mutualistic interactions are far or close to saturation ($z_i = h_i \sum_l \gamma_{il} \overline{N}_l$ either small or large), the change in γ^{LV} and α^{LV} due to a change in equilibrium abundances is small and it can be neglected, except for obligatory mutualism (see main text). Thus, we assume that the perturbation of growth rates does not modify γ^{LV} and the effective competition matrices C and that the perturbed α^{LV} is simply given by $\alpha_i^{\text{LV}}(\Delta) = \alpha_i^{\text{LV}}(\Delta = 0)\,(1 + \Delta r_i)$, where r_i are random numbers.
From the α, using Supp.Eq.(7) we compute the productivity vector as $p_i^{(\text{P})} = \alpha_i^{(\text{P})} + \sum_k G_{ik}^{(\text{P})} \alpha_k^{(\text{A})}$, where $G^{(\text{P})} = \gamma^{\text{LV(P)}} \left(\beta^{(\text{A})}\right)^{-1}$, and we obtain the perturbed productivity $p_i(\Delta) = p_i^0 + \Delta q_i$, where p_i^0 represents the unperturbed productivity, and $q_i^{(\text{P})} = \alpha^{(\text{P})} r_i + \sum_k G_{ik}^{(\text{P})} \alpha_k^{(\text{A})} r_k$ is a random variable. We then obtain $p^1(\Delta)$ by projecting the vector p onto the main eigenvector of the unperturbed effective competition matrix v^1 and we compute $\eta_i(p(\Delta))$ as

$$\begin{aligned} \eta_i(p(\Delta)) &= 1 - \frac{p_i + \Delta q_i}{v_i^1 p^1(\Delta)} \approx \left(1 - \frac{p_i^0}{p^{0,1} v_i^1}\right) + \Delta \left(\frac{q^1 p_i^0 - p^{0,1} q_i}{(p^{0,1})^2 v_i^1}\right) \\ &= \eta_i(p^0) + \Delta \frac{q^1}{p^{0,1}} \left[\eta_i(q) - \eta_i(p^0)\right] , \end{aligned} \tag{14}$$

where we neglected terms of order 2 or higher in Δ. The feasibility condition depends on the most vulnerable species, which is different in the unperturbed system $\Delta = 0$ (the species $i = v\prime$ that maximizes $\eta_i(p^0)$) and for large Δ (the species $i = v$ that maximizes $\eta_i(\Delta)$). If Δ is large enough, the most vulnerable species coincides with the species that maximizes $\eta_i(q) - \eta_i(p^0)$ and it does not change for larger Δ, so that the maximum value of $\eta_i(\Delta)$ incrases linearly as

$$\eta(\Delta) \equiv \max_i\{\eta_i(\Delta)\} \approx \eta_v(p^0) + \Delta \eta\prime(p^0) \,. \tag{15}$$

We determine Δ_c as the maximum value for which the feasibility condition $\eta(\Delta) \leq \eta^c \equiv S^{\text{eff}}/(S + S^{\text{eff}}) = (1 - \rho^{\text{eff}})/(\rho^{\text{eff}}(S-1) + 1)$ holds, i.e.

$$\Delta_\text{c}^{(\text{X})} = \frac{1}{\eta\prime^{(\text{X})}} \left(\frac{1 - \rho^{\text{eff(X)}}}{\rho^{\text{eff(X)}}(S^{(\text{X})} - 1) + 1} - \eta_v^{(\text{X})}(p^0)\right) \tag{16}$$

where we have reintroduced the superscript X (either plants or animals) for clarity. We call $\eta_v(p^0)$ the vulnerability of the unperturbed system and $\eta\prime$ the propagation of perturbations. Numerically, we compute them as

$$\eta\prime = \frac{\eta(\Delta_1) - \eta(\Delta_0)}{\Delta_1 - \Delta_0} \,, \quad \eta_v = \eta(\Delta_0) - \eta\prime \Delta_0 \,, \tag{17}$$

where $\eta(\Delta) = \max_i \overline{\eta_i(p(\Delta))}$ (the overline denotes the average with respect to perturbations) and Δ_0, Δ_1 are in the range where $\eta(\Delta)$ is linear (in the main text, we present

results with $\Delta_0 = \eta_c - 0.25$ and $\Delta_1 \eta_c + 0.25$, since we verified that Δ is linear in this range). From Eq.(14) we see that $\eta\prime$ and η_v are inversely related, since

$$\eta\prime = \frac{q^1}{p^{0,1}} \left[\eta_v(q) - \eta_v(p^0)\right] \tag{18}$$

where v is the species that maximizes $\eta_v(q) - \eta_v(p^0)$.
Supp.Eq.(16) clearly shows that the structural stability vanishes when the unperturbed vulnerability η_v approaches the critical value $\eta^c = \left(1 - \rho^{\text{eff(X)}}\right) / \left(\rho^{\text{eff(X)}}(S^{(X)} - 1) + 1\right) = S^{\text{eff}}/S + S^{\text{eff}}$.

Numerical results

We present in Supp.Fig.8 observed versus predicted values of the critical perturbation Δ_c for two values of the direct competition parameter, $\rho^{(A)} = \rho^{(P)} = 0.05$ and $\rho^{(A)} = \rho^{(P)} = 0.23$. The predicted Δ_c is proportional to $(\eta^c - \eta_v(p^0))$, with η^c a decreasing function of the effective competition parameter and, consequently, of the direct competition parameter ρ. For $\rho = 0.23$ and large CV=0.95 it holds $\eta_c < \eta_v(p^0)$ for all networks, thus the predicted Δ_c is negative. In this situation, we observe very small values of Δ_c smaller than 0.01 for all networks, in the limits of the numerical precision. These results support the prediction of our theory that Δ_c vanishes when $\eta_v(p^0)$ approaches η_c.
We then show in Supp.Fig.9 the vulnerability of the unperturbed system, $\eta_v(p^0)$, averaged over all of the networks, as a function of the variance of the distribution of the abundances. Clearly, the variance has a strong enhancing influence, i.e. the larger the variance is, the more vulnerable to extinctions is the system and the smaller is its structural stability, justifying our choice to focus our analysis on systems with narrowly distributed unperturbed abundances (we recall one again that the abundances must be narrowly distributed in species-specific units of carrying capacity, not in units of number of individuals per square kilometer).

Finally, we study how $\eta_v(p^0)$ depends on the properties of the mutualistic network such as the connectance and the nestedness. One can see from Supp.Fig.10 that $\eta_v(p^0)$ increases with the nestedness, as expected, since increasing nestedness the distribution of the mutualistic degree becomes broader, and consequently the unperturbed productivities p^0 are more broadly distributed. For fixed nestedness, $\eta_v(p^0)$ increases with the connectance only when the nestedness is large. From the figure, one can see that for CV$\approx$ 0.1 the scale of $\eta_v(p^0)$ is one order of magnitude smaller than η', justifying that its effect can be neglected in the results reported in the main text.

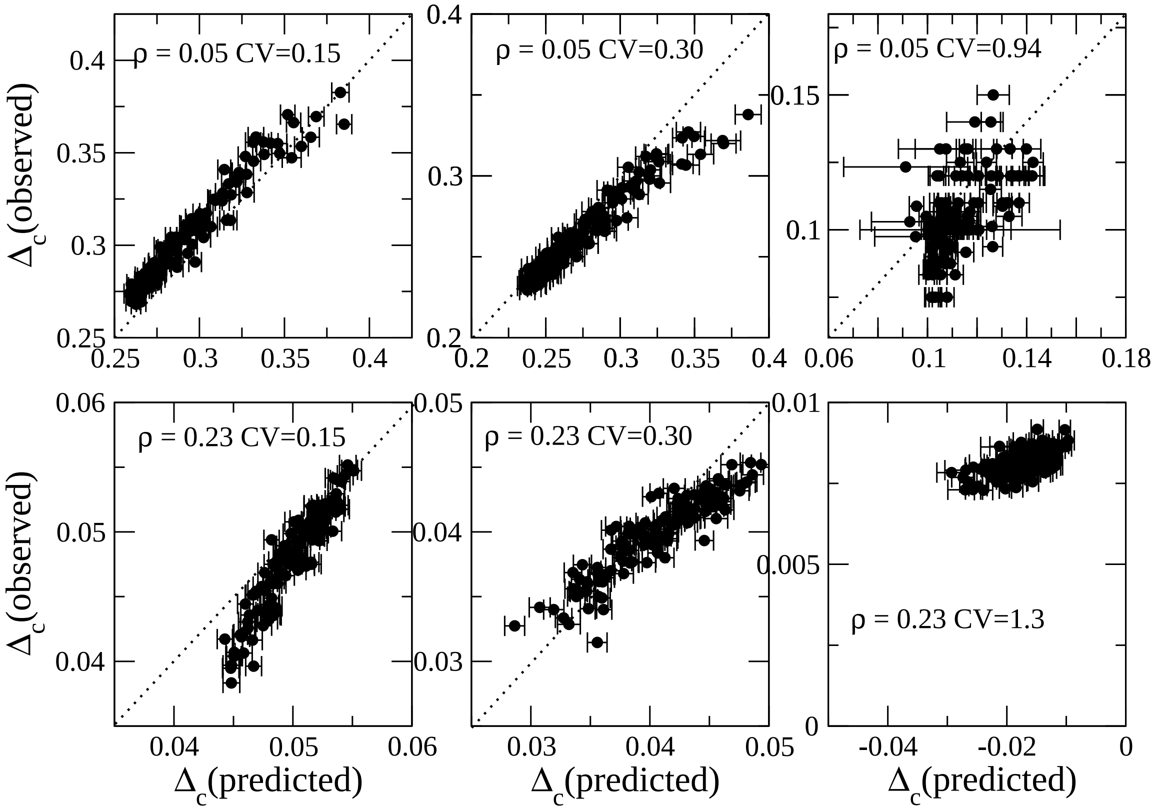

Supplementary Figure 8: **Observed versus predicted structural stability for unperturbed abundances extracted with lognormal distributions.** Top: $\rho = 0.05$. Bottom: $\rho = 0.23$. The coefficients of variation of the distributions of unperturbed abundances are CV=0.15 , CV=0.30 and CV=$0.94 - 1.3$. Each point represents one network with different connectance and nestedness, and for each of them we performed 50 independent simulations. Error bars represent the standard error of the mean over 50 realizations of the parameters and the ecological dynamics.

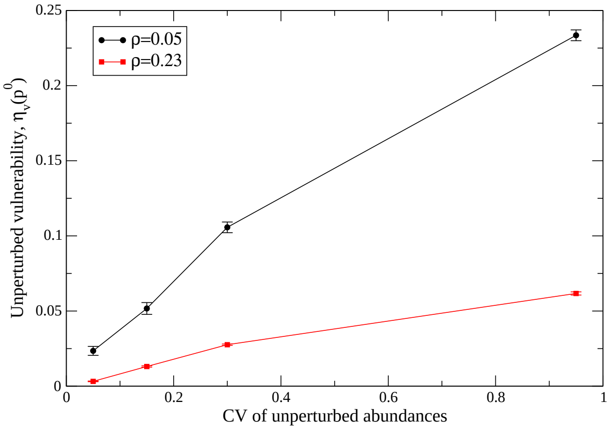

Supplementary Figure 9: **Vulnerability of the unperturbed system versus the coefficient of variation (CV) of the unperturbed distribution of abundances.** $\eta_v(p^0)$ averaged over all mutualistic networks as a function of the CV of the abundance distribution. Error bars represent the standard error of the mean over the 125 different networks.

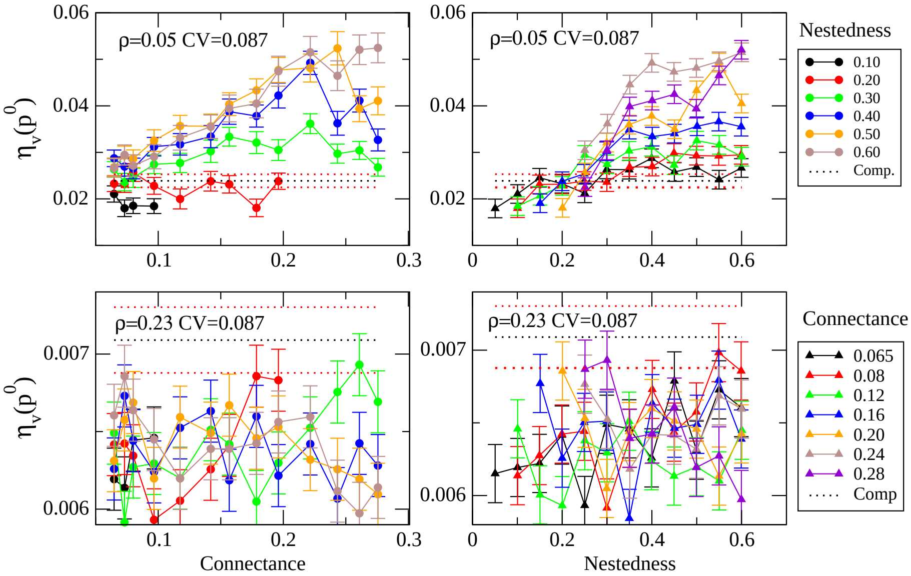

Supplementary Figure 10: **Vulnerability of the unperturbed system versus network properties.**$\eta_v(p^0)$ as a function of the connectance (left) and nestedness (right) of the mutualistic networks for CV=0.097 with $\rho = 0.05$ (top) and $\rho = 0.23$ (bottom). Each point represents one network with different connectance and nestedness, and for each of them we performed 50 independent simulations. Error bars represent the standard error of the mean over the realizations.

Supplementary Note 4: Other ecological interactions

The computations presented in the paper for mutualistic systems can be easily generalized to other types of ecological interactions. If the two groups of species compete with each other, the sign of the inter-group interaction parameter γ_0 is negative, but γ_0^2 does not change. For competitive interactions, the effective competition parameter ρ^{eff}, which depends on γ_0^2, would have the same expression as for mutualism. The qualitative interpretation is that shared competitors of the other group tend to reduce the effective competition between species of the same group exactly as shared mutualistic partners do. Also in this case we predict that there is a critical value of the direct competition parameter ρ below which competition with the other group tends to decrease the effective competition, with a beneficial effect on the structural stability, while above the critical value the opposite holds. On the other hand, competitive inter-group interactions reduce the productivity and therefore they are expected to increase the propagation of perturbations, opposite to what mutualistic interactions do, so that we expect that the connectance of the inter-group competition network tends to increase the propagation of perturbations and decrease structural stability. Also in this case, we expect that the net effect of inter-group competition on structural stability depends on the balance between the possible positive effect on ρ^{eff} and the negative effect on $\eta\prime$.

For the case of predatory interactions, the interaction is positive for one group and negative for the other group, so that we have to substitute γ_0^2 with $-\gamma_0^2$ in the expressions for the effective competition, recovering the known qualitative result that shared preys increase the effective competition between predators. The same happens for shared predators, at least if the functional response of predators is linear. The computation of the effective competition predicts that predatory inter-group interactions decrease the effective competition when the within-group competition ρ is large, with a beneficial effect on structural stability, and increase the effective competition when the within-group competition is low, opposite to what happens with mutualistic and competitive interactions. Interestingly, the predicted value of this critical competition would be the same as for mutualistic and competitive interactions. On the other hand, predatory inter-group interactions increase the productivity of predators but decrease the productivity of preys, thus they are expected to have opposite effects on the propagation of perturbations of predators and preys. We expect that the net effect on structural stability of the connectance of the inter-group predatory network depends on the balance between these two effects, and changes depending on the regime of parameters.

In summary, we expect that for weak within-group competition, inter-group interactions of mutualistic and competitive type tend to decrease the effective competition, while inter-group interactions of predatory type tend to increase it, and that the opposite holds for strong direct competition.

Supplementary References

[1] Bastolla, U., Fortuna, M.A., Pascual-García, A., Ferrera, A., Luque, B. & Bascompte, J. The architecture of mutualistic networks minimizes competition and increases biodiversity, *Nature* **458,** 1018-1020 (2009).

[2] James, A., Pitchford, J.W. & Plank, M.J. Disentangling nestedness from models of ecological complexity, *Nature* **487,** 227-230 (2012).

[3] Pascual-García A., Ferrera A. and Bastolla U., Comment to "Disentangling nestedness from models of ecological complexity" http://arxiv.org/abs/1409.1683

[4] Bastolla, U., Lässig M., Manrubia, S.C. and Valleriani A. Biodiversity in model ecosystems, I: coexistence conditions for competing species. *J. Theor. Biol.* **235,** 521-530 (2005).

[5] Meyer, C. Matrix analysis and applied linear algebra. SIAM, ISBN 0-89871-454-0 (2000).

[6] Ferrera A, Pascual-García A and Bastolla U. Effective competition determines the global stability of model ecosystems. Theor. Ecol., in the press (2016).

[7] Pascual-García A, Ferrera A and Bastolla U . Effective competition determines the structural stability of model ecosystems. Preprint.

[8] Goh B.S. Global stability in many-species systems. The American Naturalist 111, 135-143 (1977).

[9] Rohr RP, Saavedra S, Bascompte J Ecological networks. On the structural stability of mutualistic systems. Science 345:1253497 (2014).
